# Antibiotics adsorb to polyester microplastic fibers and alter bacterial communities in stream mesocosms

**DOI:** 10.3389/fmicb.2026.1847208

**Published:** 2026-07-23

**Authors:** Ryan T. Krantz, Sathaporn Onanong, Daniel D. Snow, Timothy J. Hoellein, John J. Kelly

**Affiliations:** 1 Department of Biology, Loyola University Chicago, Chicago, IL, United States; 2 Water Sciences Laboratory, University of Nebraska-Lincoln, Lincoln, NE, United States

**Keywords:** adsorption, antibiotics, bacteria, freshwater, mesocosm, microplastic, polyester, resistance

## Abstract

Antibiotics and microplastics (MPs) are anthropogenic contaminants that co-occur in wastewater and wastewater-impacted surface waters. Within these environments antibiotics can select for resistant taxa and genes, altering the composition of bacterial communities and potentially contributing to the spread of antibiotic resistance genes (ARGs). Antibiotics can also adsorb to MPs, resulting in high antibiotic concentrations on MP surfaces. We hypothesized that MPs exposed to antibiotics in a freshwater environment would adsorb antibiotics and that this interaction would alter the composition of bacterial biofilms colonizing the MP surfaces, including selecting for antibiotic resistant taxa and increased abundance of ARGs. We tested these hypotheses with a stream mesocosm experiment that included polyester fibers, a mixture of eight antibiotics at two environmentally relevant concentrations, and an inoculum of bacteria from an urban stream. The addition of antibiotics to the streams resulted in significant adsorption of ciprofloxacin, sulfamethoxazole, and trimethoprim to the MPs (*p* < 0.001), with ciprofloxacin showing the highest adsorption. The addition of antibiotics to the streams also significantly changed the composition of the bacterial communities on the MPs and in the water (*p* < 0.05), including significant increases in the abundance of bacterial orders linked to antibiotic metabolism and resistance (e.g., *Sphingobacteriales*, *Caulobacterales*, and *Xanthomonadales*). However, the abundance of two ARGs, *mexB* and *tetA*, did not vary with antibiotic dosage, and the abundance of an integrase gene, *intI1*, decreased with antibiotic dosage. These results confirmed our prediction that antibiotics would adsorb to MPs in freshwater and alter the composition of particle-associated and planktonic bacterial communities, but the presence of antibiotics did not increase the abundance of ARGs.

## 1 Introduction

The discovery of antibiotics in the mid-20th century revolutionized the treatment of disease, but the implementation of these new chemotherapeutic compounds coincided with the nearly simultaneous development of resistance among bacterial pathogens (Abraham and Chain, 1940; Marston et al., 2016). Despite widespread research, international policy regarding antibiotic resistance as a global threat only emerged within the last 30 years (Overton et al., 2021), and use of prescription antibiotics and antimicrobial consumer products continues to increase worldwide (Boeckel et al., 2014; Klein et al., 2024).

Incompletely metabolized oral antibiotics and antimicrobials in products like soaps, wipes, and disinfectants routinely enter wastewater (Larsson and Flach, 2022; Merchel Piovesan Pereira and Tagkopoulos, 2019), resulting in antibiotic concentrations in wastewater ranging from 1 to 10 μg L^−1^ (Supplementary Table S1). Wastewater treatment systems do not completely remove most antibiotics (Watkinson et al., 2007), resulting in release of these compounds into surface waters via effluent (Lorentz et al., 2025) and leading to antibiotic concentrations in urban streams ranging from 0.1 to 1.0 μg L^−1^ (Supplementary Table S1). Antibiotics can also enter the environment via direct release of untreated wastewater caused by combined sewer overflows, leaking infrastructure, or lack of wastewater treatment systems (Rosi-Marshall and Royer, 2012). Because *in situ* antibiotic concentrations are generally sub-inhibitory (Chow et al., 2021), antibiotics in wastewater create an evolutionary pressure that can select for antibiotic resistant taxa and antibiotic resistance genes (ARGs). As global consumption of antibiotics rises, so will the prevalence of antibiotics in wastewater and freshwater systems, further selecting for antibiotic resistance in microbial communities.

Plastic particles smaller than 5 mm in diameter (microplastics, MPs) are also ubiquitous contaminants in wastewater, largely stemming from MP fibers shed from synthetic textiles (Acharya et al., 2021). These particles are not completely removed from wastewater, and treated effluent is a point source of MP fibers to aquatic habitats (Kelly et al., 2021; McCormick et al., 2016; Simon et al., 2018). MPs in wastewater may also enter the environment during combined sewer overflows (Di Nunno et al., 2021) or when wastewater infrastructure is ineffective, damaged, or lacking (Woodward et al., 2021). Over the coming decades, leakage of MPs into the environment is projected to increase three-fold (OECD, 2022), indicating that MPs will persist as environmental contaminants.

MPs in wastewater and in the environment can adsorb contaminants like antibiotics to their surfaces due to hydrophobic interactions, hydrogen bonding, electrostatic interactions, and pore filling (Stapleton et al., 2023), and contaminant concentrations on MPs may be orders of magnitude greater than in the surrounding water (Li et al., 2018; Mato et al., 2001). Unlike biodegradable substrates in freshwater, MPs are durable and persistent in the environment (Vincent and Hoellein, 2021), and because many MPs are also buoyant (Summers et al., 2023) they may travel great distances before settling. MP particles therefore may act as transport vectors for adsorbed compounds (Atugoda et al., 2021; Chand et al., 2025). MPs in wastewater and in the environment are also readily colonized by microbial communities, creating opportunities for these microbes to interact with high concentrations of adsorbed contaminants (Krantz et al., 2026). Selection pressure from MP-adsorbed antibiotics has the potential to promote ARGs within these communities. For example, ARGs can be propagated by horizontal gene transfer (HGT), via mechanisms including conjugation, transformation, and transduction (Lorenz and Wackernagel, 1994; Thomas and Nielsen, 2005). HGT is especially prevalent in biofilm communities due to the close proximity and extended retention of diverse bacteria within them (Madsen et al., 2012).

The potential for interactions between antibiotics and microbial communities on MP surfaces has only recently begun to be explored (Muñoz-Palazon et al., 2025; Song et al., 2026; Nnadozie et al., 2025), and studies explicitly linking antibiotic adsorption to microbial community impacts are needed. In this study we address this knowledge gap by investigating the interactions between antibiotics and microbial biofilm communities on MP surfaces. We designed an experiment incorporating environmentally relevant concentrations of eight common antibiotics and MP fibers using artificial stream mesocosms that simulate the movement, dynamics, and gas exchange of an urban stream. The two antibiotic concentrations implemented in the study represent concentrations reported in wastewater and urban streams and can therefore provide insight into the magnitude of the effects when untreated wastewater enters the environment. We hypothesized that addition of antibiotics to streams would result in adsorption of antibiotics to MP surfaces, that the addition of antibiotics would alter the taxonomic composition of bacterial communities in the water and on MP surfaces, and that ARGs would become more abundant in the water and on MP surfaces with elevated antibiotic concentrations.

## 2 Materials and methods

### 2.1 Stream mesocosms

We used recirculating fiberglass stream mesocosms (Drury et al., 2013) to investigate the effects of MPs and antibiotics on bacterial communities. Each 4 m × 15.5 cm × 15 cm stream contained a metal rotating paddle wheel connected to a motor that circulated approximately 60 L of water at an average velocity of 0.212 m s^−1^. Streams were located in a greenhouse on the campus of Loyola University Chicago. The stream experiment was initiated on November 6, 2024, so the streams were exposed to a photoperiod of approximately 10 h daylight per day. To prevent excessive algal growth within the streams, they were completely covered with WeedBlock landscape fabric (Easy Gardener, Waco, TX), blocking 92% of visible light (manufacturer’s specification).

### 2.2 Antibiotic treatments

We selected the concentration of antibiotics that we added to the streams based on a survey of published studies that measured antibiotic concentrations in wastewater influent and effluent, and in freshwater streams (Supplementary Table S1). Maximum reported antibiotic concentrations in wastewater influent and effluent were ~5,000 ng L^−1^, and concentrations in urban streams were ~500 ng L^−1^. We based the “high dose” and “low dose” experimental treatments implemented in this study on these concentrations, respectively. Both of these concentrations are sub-inhibitory (Chow et al., 2021). A third treatment contained no antibiotics and served as a control. We applied these treatments to four replicate streams each (12 total streams), which were arranged to control for variability resulting from the position of individual streams within the greenhouse (Supplementary Figure S1).

### 2.3 Preparation of microplastics

We chose polyester fibers as the representative MP for this study. Due to their prevalent use in synthetic textiles (Textile Exchange, 2024), polyester comprises the largest proportion of MP fibers in wastewater effluent and in the environment (Browne et al., 2011). Additionally, polyester fibers adsorbed significantly more antimicrobials than other polymers in a previous study (Krantz et al., 2026). We added fibers to each stream to achieve a concentration of 500 MP L^−1^, which is representative of the maximum reported values in wastewater (Conley et al., 2019).

We prepared MP fibers from recycled 100% polyester yarn (SKU: 126-107 U; Lion Brand Yarn Company, Rincon, GA). To confirm the polymer composition of this yarn, we performed micro-Fourier transform infrared (FT-IR) spectroscopy using a PerkinElmer Spectrum Two Spectrometer (Waltham, MA, USA). We compared spectra collected between 450–4,000 wavelengths cm^−1^ to a reference library of polymer spectra provided by PerkinElmer using Spectrum 10 software (version 10.6.2), which matched the yarn to poly(ethylene terephthalate) with a correlation of 0.886 (Supplementary Figure S2). We then prepared MP fibers by cutting the yarn into ~3 mm lengths using a razor and wood block. We placed approximately 30 fibers on gridline filter paper and examined them with a dissecting microscope to measure fiber dimensions: average length = 3.21 ± 0.12 mm; width = 0.045 mm.

We generated a fiber count × mass regression to determine the mass of fibers needed to achieve our target MP concentration for each of the streams (Supplementary Figure S3). We first cut the yarn into 3 separate samples spanning a weight gradient then cut each sample into fibers (as described above) and suspended the fibers in 50 mL 95% ethanol in a sterile glass flask. We used ethanol rather than water to ensure homogeneous suspension of the fibers. For each sample, we removed three 5 mL subsamples, collected the MPs from each subsample on gridline filter paper via vacuum filtration, and manually counted the MPs using a dissecting microscope. From these data we generated a count × mass regression which we used to prepare samples of ~30,000 fibers for each stream. This generated our target concentration of 500 MP L^−1^ in each 60 L stream, representative of MP concentrations reported in untreated wastewater (Conley et al., 2019). We prepared four additional samples of ~30,000 fibers to serve as pre-incubation controls to measure pharmaceutical concentrations and analyze microbial communities on the MPs prior to their addition to the streams.

### 2.4 Preparation of antibiotic treatments

We used eight antibiotics in this study: amoxicillin, azithromycin, ciprofloxacin, doxycycline, gentamicin, nitrofurantoin, sulfamethoxazole, and trimethoprim (Supplementary Table S2). We selected these compounds based on a combination of factors, including prescription rates in the United States, reported concentrations in wastewater and anthropogenically-impacted streams, potential to adsorb to MP surfaces, and to encompass a range of antibiotic classes. We purchased all antibiotics as powders from Thermo Fisher Scientific (Ward Hill, MA) and stored them in the dark at 4 °C prior to the experiment. The day before the experiment, we prepared individual stock solutions of each antibiotic with sterile deionized (DI) water, mixed them thoroughly with magnetic stir bars, then stored them in the dark at 4 °C overnight. The day of the experiment, we thoroughly mixed the stock solutions via magnetic stir bars then combined them to create eight 1 L antibiotic “cocktails” containing all eight antibiotics. We prepared four cocktails containing 30 μg L^−1^ of each antibiotic for the low dose streams and four cocktails containing 300 μg L^−1^ of each antibiotic for the high dose streams. When added to the 60 L streams, these cocktails resulted in final experimental concentrations of 500 ng L^−1^ and 5,000 ng L^−1^, respectively.

### 2.5 Microbial inoculum

We collected water and suspended sediment from the North Branch of the Chicago River at Harms Woods Forest Preserve (coordinates: 42.062651, −87.771024) in plastic carboys to inoculate the mesocosms with natural microbial communities (Drury et al., 2013). We measured water quality metrics (Supplementary Table S3) at three adjacent locations in the river at the time of collection (ProDSS multiparameter meter, YSI, Yellow Springs, OH). We transported the carboys to the laboratory and stored them at 4 °C overnight prior to the start of the experiment. We collected four additional 1 L water samples from the river for nutrient chemistry analysis, transported these samples to the laboratory in a cooler, and stored them at −20 °C until analysis was performed. After thawing, we filtered one 50 mL subsample from each bottle through a separate 0.45 μm Nalgene disposable filter (Thermo Fisher Scientific, Waltham, MA) then processed each filtered sample with a 940 Professional IC Vario chromatograph (Metrohm, Riverview, FL) to measure ionic concentrations (Supplementary Table S4).

### 2.6 Stream experiment

Prior to the start of the experiment, we filled each stream with a total of 60 L of water. We first added dechlorinated tap water according to experimental treatment: high dose streams (*n* = 4) and low dose streams (*n* = 4) received 53 L, and control streams (*n* = 4) received 54 L. We turned on the motors powering the rotating paddle wheels and let them run for 24 h to aerate and dechlorinate the water. On the day the experiment started, we added river water inoculum, MP fibers, and antibiotic cocktails to each stream. First we added 6 L of Chicago River water to each stream to provide an inoculum of natural microbial communities. Next, we added to each stream 30,000 MP fibers suspended in 10 mL 95% ethanol. We used ethanol rather than water to homogeneously suspend the MPs. We expected that exposure of the MPs to ethanol would significantly reduce the microbial load on MP surfaces prior to their addition to the streams, ensuring that bacteria detected on the MPs after the experiment predominantly originated from the stream water. We swirled each flask to suspend the MPs and slowly emptied the contents into the stream. Finally, we added 1 L antibiotic cocktails (described above) to the high dose and low dose streams. During the experiment, we monitored the streams daily for changes in water level and replenished each stream with municipal water that had dechlorinated for at least 24 h. On average, we added 1 L water per day to each stream to compensate for evaporative loss.

Using extra water from the Chicago River, we collected microbes from six 50 mL subsamples to analyze the community composition of the initial inoculum. We collected microbes by filtering each 50 mL subsample of river water through a 0.22 μm Sterivex GP sterile filter unit (Merck KGaA, Darmstadt, Germany) using a 30 mL Luer lock syringe. Following a published protocol for isolating planktonic microbes from environmental water samples (Crump et al., 2003), we removed the filters from the canisters, cut them into fragments using a sterile razor, placed them in a sterile 2 mL screwcap microcentrifuge tube, and stored them at −20 °C.

### 2.7 Collection of samples

We ended the experiment after 18 days and collected water and MP samples from each stream. We siphoned water from each stream using 80 mm diameter Tygon scientific tubing (Saint-Gourbain, Courbevoie, France) and passed the water through a 125 μm gauge sieve to collect MP fibers. Before sampling the water from each stream, we rinsed and sterilized the sieve with 70% ethanol. After filtering, we removed large debris such as plant matter or non-fiber plastics from the sieve using forceps sterilized with 70% ethanol. For each stream, we used the forceps to place a ~ 50 μL subsample of the collected MP fibers into a sterile 2 mL screwcap microcentrifuge tube then stored them at −20 °C for subsequent microbial analysis. We used the forceps to transfer the remainder of the MPs from the sieve to a sterilized glass beaker covered with aluminum foil then suspended them in 10 mL autoclaved DI water. We also processed the four pre-incubation control MP samples (described in section 2.3) in this manner. We suspended each control MP sample in 10 mL of 95% ethanol, collected the fibers with the 125 μm gauge sieve, and subsampled the fibers for microbial community and antibiotic analysis, as described above. In addition to collecting MPs from each stream, we collected 1 L of the stream water flowing through the sieve for microbial community and nutrient chemistry analyses. We collected microbes from this water using 0.22 μm Sterivex GP sterile filter units (as described in section 2.6) then separately stored the filters and remaining water at −20 °C for subsequent analyses.

### 2.8 Antibiotic analysis

We measured antibiotics adsorbed to both the control MPs and the MPs collected from each stream. For each sample, we vacuum filtered (300 mL Pall filter funnel) MPs suspended in sterile DI water onto a pre-weighed filter (Whatman ME 25/21 ST 0.45 μm mixed cellulose ester membrane gridline filter, Cytiva, Marlborough, MA). We examined each filter using a dissecting microscope to confirm the presence of MP fibers and removed any visible organic debris using forceps. We then weighed the filter and determined the mass of MP fibers by subtraction. We placed the filters containing MPs into glass scintillation vials and stored them at −20 °C. We shipped all MP samples to the Water Sciences Laboratory at the University of Nebraska-Lincoln to measure adsorption of antibiotics to the MPs. We did not measure antibiotic concentrations in the stream water.

We extracted and analyzed adsorbed antibiotics by liquid chromatography tandem mass spectrometry (LC–MS/MS) using a modified version of EPA Method 1694 Group 1 pharmaceuticals (USEPA 2007; Ferrer et al., 2010). Briefly, we transferred massed microplastic samples, ranging from 0.0128 g to 0.0469 g, into 50 mL centrifuge tubes and mixed with 2 g of combusted high purity silica sand, 15 mL McIllvaine buffer (pH = 4.5), 20 mL acetonitrile, and fortified with 100 μL of a 0.1 ng μL^−1^ surrogate solution (^13^C_3_-atrazine, ^13^C_3_-deethylatrazine, oleandomycin and orphenadrine). We shook the capped centrifuge tubes for 30 min in a wrist-action shaker and centrifuged them at 3,200 rpm. We decanted the extraction liquid into a 150 mL glass evaporation tube (RapidVapN2, Labconco, St. Joseph, MO). We repeated the extraction using an additional 15 mL of buffer and 20 mL of acetonitrile, combined the extraction liquid with the first portion in the evaporation tube, and evaporated the contents at 40 °C using nitrogen and agitation to approximately 20 mL. We added ultrahigh purity reagent water to bring the volume to 50 mL then slowly passed the aqueous extract through a preconditioned solid phase extraction cartridge (HLB, 200 mg, Waters Corporation, Milford, MA). We eluted the preconcentrated compounds from the cartridge using 5 mL of acetonitrile into a 10 × 75 mm glass culture tube, concentrated the solution to 500 μL under nitrogen, and filtered the solution using a 0.2 μm nylon syringe filter into a second culture tube to remove any remaining particulate. After spiking with 200 μL of labelled internal standard mix (0.1 ng μL^−1^ of ^13^C_6_-sulfamethazine, ^13^C_3_-caffeine, salinomycin, ^13^C-^15^N-acetominophen, ^13^C_6_-carbamazipine, ^13^C_6_-sulfametoxazole, ^13^C_6_-thiabendazole, ^13^C_6_-erythromycin, cotininefluoxitine-d6, ^13^C_3_-trimethaprim, ^13^C_3_-^15^N-ciprofloxicin), we vortexed the contents, evaporated to 40 μL, vortexed again with 160 μL ultrahigh purity reagent water, and transferred the liquid to autosampler vials fitted with 300 μL silane-treated glass inserts. We then analyzed the extracts by multi-reaction monitoring LC–MS/MS.

We analyzed all extracts on a Xevo TQS Micro triple quadrupole mass spectrometer interfaced to an Acquity H-Class ultrahigh pressure liquid chromatography system with a Uni-Spray™ ionization source (Waters Corporation, Milford, MA USA). We modified the instrumental conditions to include amoxicillin and nitrofurantoin, but doxycycline and gentamycin were incompatible with the extraction and instrumental conditions and could not be quantified. Details of the instrumental conditions, method sensitivity and validation experiments are included in the supplemental materials (Supplementary Tables S5–S7). We conservatively adjusted the method detection limits used in statistical tests upward by 20 times (equivalent to a 0.050 g sample size) to account for the small microplastic sample size which averaged 0.026 g. Low and variable recovery for some analytes such as ampicillin (23 ± 42%) and fluoxetine (1.0 ± 55%) observed in the 5 ng g^−1^ fortified reagent grade polyethylene matrix likely reflect non-recoverable and irreversibly bound target compounds in this matrix. Low recovery may also reflect extractable plasticizers leading to suppression of ionization and matrix effects. Surrogate recovery in actual sample extracts averaged 60% for orphenadrine, 77% for oleandromycin, 94% for ^13^C_3_-atrazine, and 96% for ^13^C_3_ deethylatrazine. We used method blanks and fortified blanks using 1 g polyethylene powder as quality controls.

### 2.9 Microbial community analysis

We isolated microbial DNA from all MP and water samples using the DNeasy PowerBiofilm Kit (Qiagen, Hilden, Germany). We amplified partial bacterial 16S rRNA genes from these extractions via polymerase chain reaction (PCR) using primers 515F and 806R (Caporaso et al., 2012). Thermocycling parameters were as follows: initial denaturing at 95 °C for 5 m, 28 cycles consisting of 30 s denaturing at 95 °C, 45 s annealing at 55 °C, and 30 s extension at 72 °C, and a final 7 min extension at 72 °C. We confirmed amplification of 16S genes via agarose gel electrophoresis. We were not able to amplify 16S genes from the pre-incubation control MP samples exposed to 95% ethanol, indicating that ethanol exposure reduced the microbial load on the pre-incubation MPs below the detection limit of PCR. Amplicons were sequenced by the Genomics and Microbiome Core Facility at Rush University (Chicago, IL) in 2 × 250 paired-end format using the MiSeq platform (Illumina, San Diego, CA, USA). Raw sequence data are accessible via the National Center for Biotechnology Information Sequence Read Archive with accession number PRJNA1448104.

We processed the 16S rRNA sequences with mothur v.1.48.0 (Schloss et al., 2009) following the MiSeq Standard Operating Procedure (Kozich et al., 2013). We demultiplexed and assembled paired-end reads and removed sequences with ambiguities or homopolymers larger than 8 bases. We aligned the sequences to the SILVA-compatible database in mothur, then identified and removed chimeras using UCHIME (Edgar et al., 2011). We classified the sequences according to the RDP training set formatted for mothur (v.19) and removed non-bacterial sequences. We clustered sequences with at least 97% identity into operational taxonomic units (OTUs), hereafter referred to as species. We rarefied the data to the number of sequences in the sample with the fewest sequences (50,056) to account for uneven distribution of sequences among samples. We estimated coverage of the bacterial communities from each sample (Good, 1953), which indicated that coverage averaged 96.8% with a range of 93.3 to 98.4%. We also used mothur to determine species richness (total observed species), alpha diversity (Shannon diversity index), and beta diversity (based on the Bray-Curtis index) at the OTU level.

### 2.10 Quantification of antibiotic resistance genes

We screened DNA isolated from MP and water samples for the presence of ten ARGs via end-point PCR and agarose gel electrophoresis. Primer and thermocycling information for all target genes are listed in Supplementary Table S8. All primer sets amplified their respective ARGs from reference DNAs containing the gene of interest, but only three primer sets amplified ARGs from the experimental samples: *intI1*, *mexB*, and *tetA*. For these three genes, we determined gene abundance via quantitative PCR (qPCR) with a QuantStudio 3 Real-Time PCR System (Thermo Fisher Scientific, Waltham, MA). We prepared standard dilution series of 10^8^–10^2^ copies of each target gene from reference DNAs for each primer set, testing for quality prior to quantification of experimental samples (Supplementary Table S9). Reaction mixtures included 12.5 μL QuantiTect SYBR green PCR master mix (Qiagen, Hilden, Germany), 9 μL nuclease free water, 1.25 μL per primer, and 1 μL template DNA. Thermocycling included an initial denaturing step at 95 °C for 15 min, 40 cycles consisting of denaturing at 95 °C for 45 s, annealing for 45 s, extension at 72 °C for 45 s, and imaging at 78 °C for 12 s, and a final melt curve step. We quantified the full set of experimental samples via qPCR with validated standards using the same primers and parameters. We quantified 16S rRNA gene copies in all samples using primers 515F and 806R (Bates et al., 2011) and annealing at 55 °C.

### 2.11 Data analysis and statistics

We completed initial statistical analysis of bacterial sequence data within the mothur pipeline: we assessed bacterial community beta diversity by ordinating a Bray–Curtis dissimilarity matrix (Bray and Curtis, 1957) using nonmetric multidimensional scaling (NMDS), and we determined the significance of differences between communities using analysis of molecular variance (AMOVA; Excoffier et al., 1992), an analog of traditional ANOVA.

We visualized data from mothur using R Statistical Software (v4.3.1; R Core Team, 2023) with ggplot2 (v3.5.2; Wickham, 2016), ggpubr (v0.6.1; Kassambara, 2023), dplyr (v1.1.4; Wickham et al., 2023), and tidyr packages (v1.3.1; Wickham et al., 2024), and analyzed the data using statistics from base R and the car package (v3.1–3; Fox and Weisberg, 2019). We identified statistically significant differences between group means via analysis of variance (ANOVA, Fisher, 1992), testing for assumptions of ANOVA using Levene’s test for homogeneity of variance (Levene, 1960) and the Shapiro–Wilk test for normality (Shapiro and Wilk, 1965) in addition to visually assessing the data using cell mean vs. standard deviation and normal probability plots. When assumptions were not met, we transformed the data using the Box-Cox transformation (Box and Cox, 1964) and reevaluated; all reported results represent data that met these assumptions. We further investigated significant ANOVA results (*α* = 0.05) using the Tukey HSD multiple comparisons test (Tukey, 1949) to determine significant pairwise differences.

We quantified ARGs by normalizing gene abundance to copies of the bacterial 16S rRNA gene. Proportional data were transformed via arcsine square root transformation to better meet the assumptions of ANOVA before further statistical analysis.

## 3 Results

### 3.1 Adsorption of antibiotics to MPs

Adding a cocktail of eight antibiotics at environmentally relevant concentrations to the mesocosms resulted in the adsorption of several antibiotics to MP surfaces (Table 1). Specifically, the concentrations of ciprofloxacin, trimethoprim, and sulfamethoxazole were significantly higher on MPs collected from the high dose mesocosms compared to MPs from all other treatments (Tukey’s HSD: *p* ≤ 0.04), and concentrations of ciprofloxacin and trimethoprim were significantly higher on MPs collected from the low dose mesocosms compared to pre-incubation MPs and MPs collected from control mesocosms (*p* ≤ 0.001). In contrast, amoxicillin, azithromycin, and nitrofurantoin were not detected on any MPs.

**Table 1:** Concentrations of antimicrobials (ng g^−1^) adsorbed to polyester MPs according to antimicrobial treatment.

Antibiotic	Pre-incubation	Control	Low dose	High dose	ANOVA *p*-value
Amoxicillin	ND	ND	ND	ND	-
Azithromycin	ND	ND	ND	ND	-
Ciprofloxacin	164 ± 60 a	44 ± 19 a	5,602 ± 1751 b	24,403 ± 3,497 c	< 0.001
Nitrofurantoin	ND	ND	ND	ND	-
Sulfamethoxazole	BD ab	BD a	BD b	47 ± 4 c	< 0.001
Trimethoprim	BD a	BD a	65 ± 33 b	754 ± 139c	< 0.001

Numerical values represent average concentrations within treatments ± standard error (*n* = 4). Different lower-case letters represent significantly different groups. Non-detected compounds (ND) and detected values below the adjusted detection limit of the assay (BD) are also reported.

### 3.2 Effects of antibiotic dosage on bacterial communities

**Figure 1:**
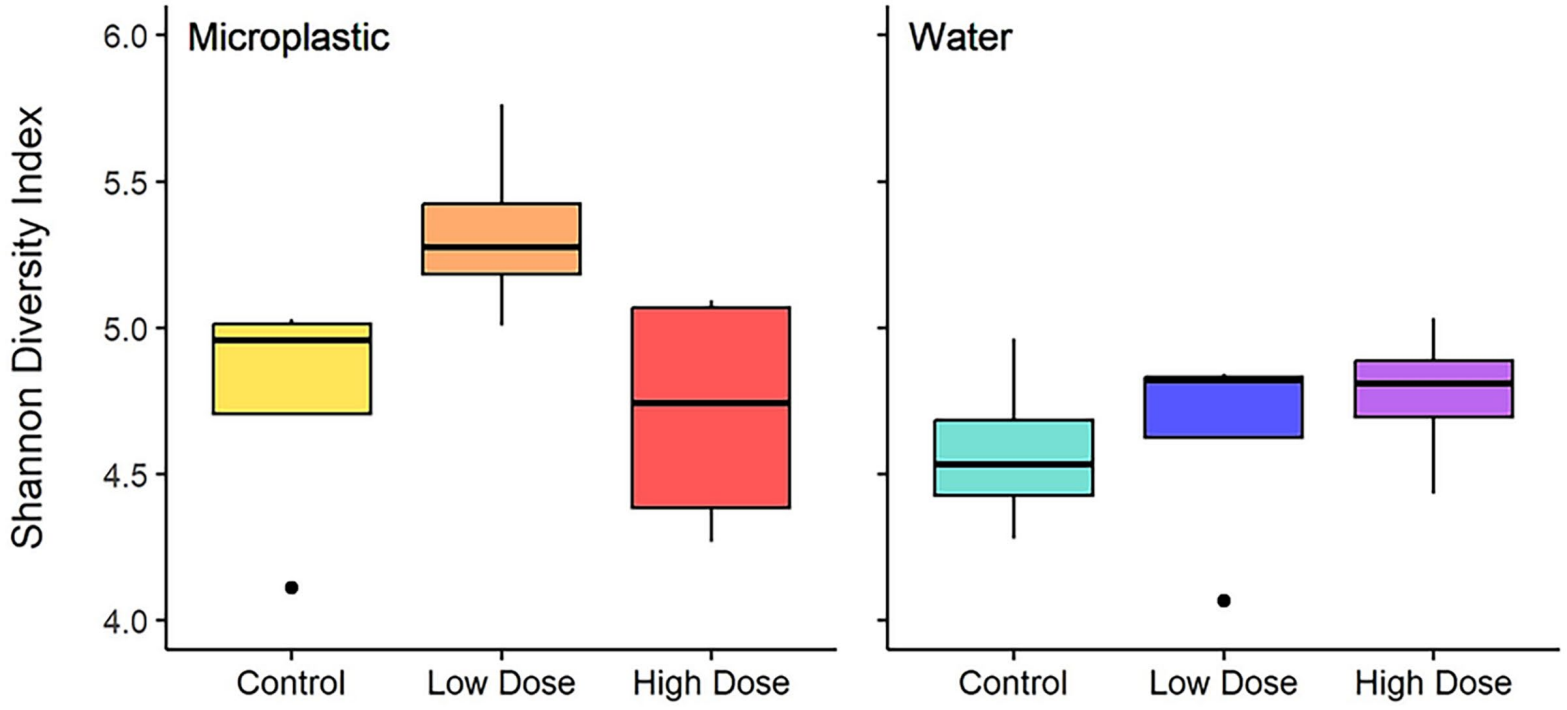
Shannon diversity index of bacterial communities on MPs and in water (*n* = 4). ANOVA indicated no significant differences in diversity according to antibiotic dosage or habitat type (MP or water).

**Figure 2:**
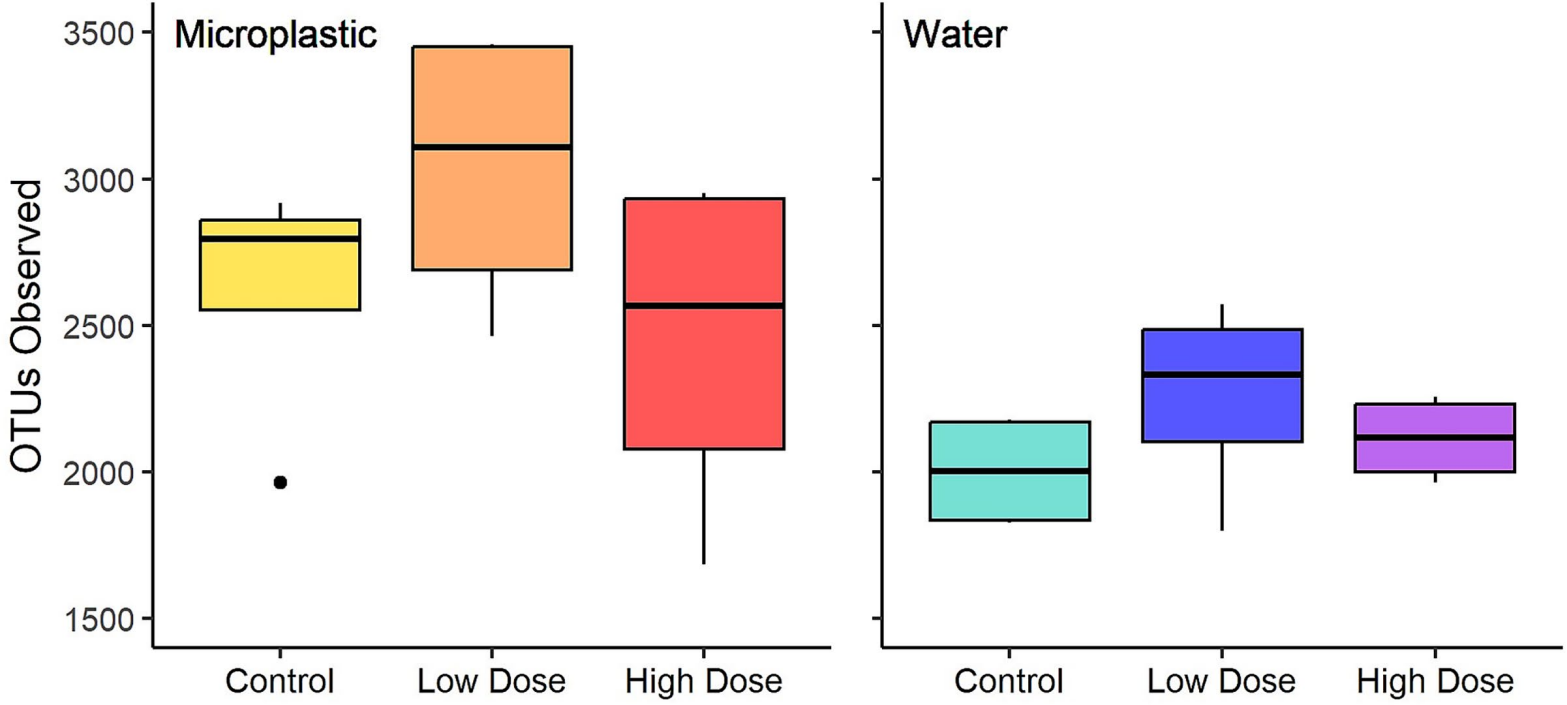
Total bacterial species (OTUs) observed within communities on MPs and in water (*n* = 4). ANOVA indicated significantly more species on MP samples compared to water samples (*p* = 0.003), but no statistical differences according to antibiotic dosage.

Alpha diversity of bacterial communities on MPs and in water was not affected by antibiotic dosage, either in terms of overall diversity (Figure 1) or number of bacterial species (Figure 2). Shannon diversity was statistically equivalent among all samples, but MP samples contained significantly more species than water samples across all treatments (ANOVA: *F*_0.05, 1, 22_ = 11.24; *p* = 0.003).

**Figure 3:**
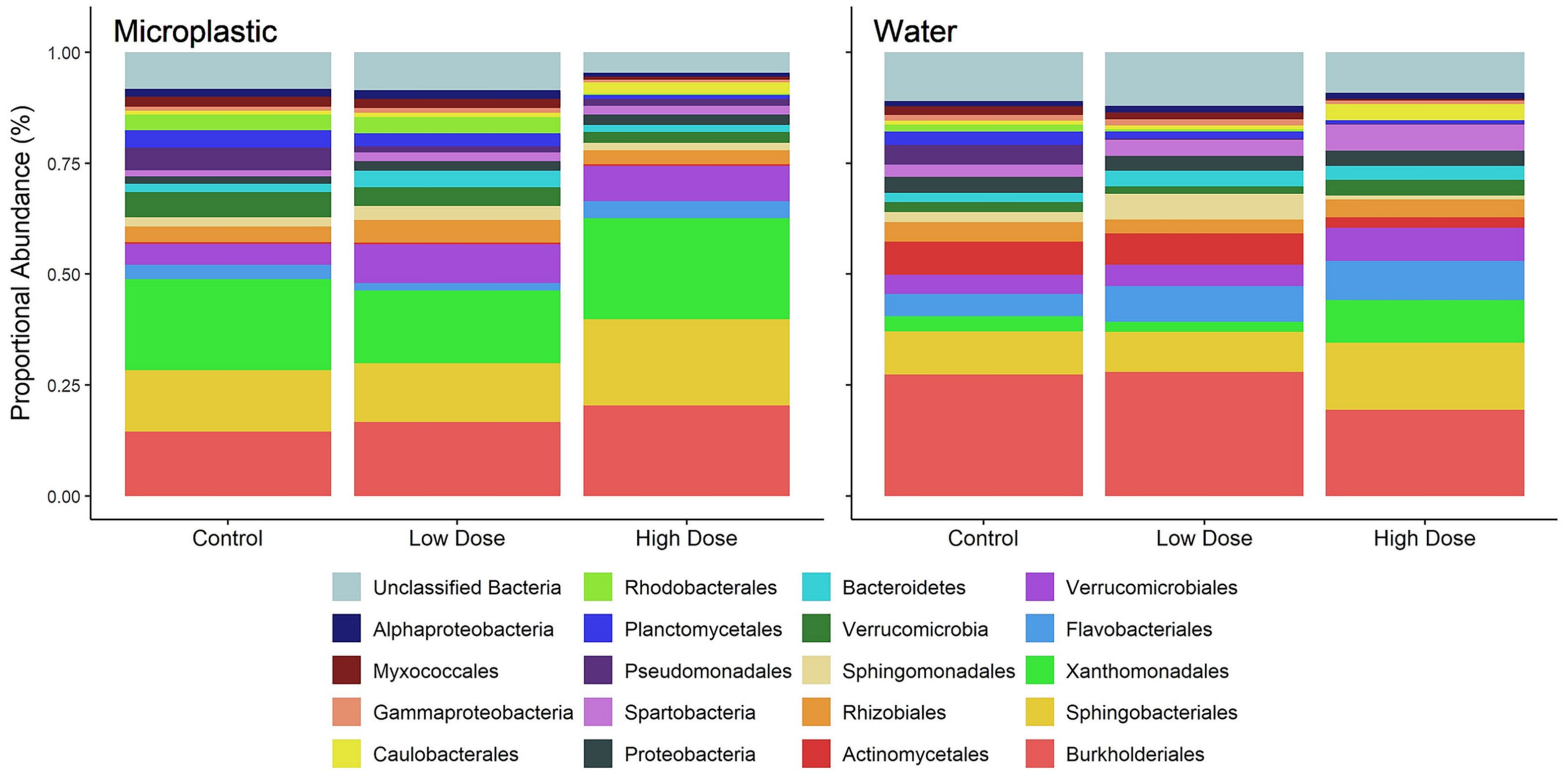
Average proportional abundance of the 20 most abundant bacterial orders on MPs and in water, according to antibiotic dosage (*n* = 4). Orders were identified to the lowest possible taxonomic rank.

Although alpha diversity did not differ according to antibiotic dosage, several bacterial orders varied among treatments, particularly in high dose streams (Figure 3; Table 2). *Caulobacterales* and *Sphingobacteriales* were more abundant on high dose MP samples, whereas *Planctomycetales*, *Rhodobacterales*, and *Verrucomicrobia* were less abundant compared to low dose and control samples. *Caulobacterales* and *Sphingobacteriales* were also more abundant in high dose water samples, in addition to *Xanthomonadales* and *Spartobacteria*, whereas *Rhodobacterales* and *Sphingomonadales* were relatively less abundant.

**Table 2:** Significant differences in the abundance of bacterial orders on MPs and in water, according to antibiotic dosage. Numerical values represent average abundance within treatments ± standard error (*n* = 4). Superscript numbers indicate taxa that differed in both habitat types, and different lower-case letters represent significant differences in taxa across treatment groups.

Bacterial order	Control	Low dose	High dose	ANOVA *p*-value
Microplastic				
*Caulobacterales* ^1^	389 ± 88 a	396 ± 9 a	1167 ± 114 b	<0.001
*Planctomycetales*	1775 ± 171 a	1287 ± 192 a	394 ± 153 b	<0.001
*Rhodobacterales* ^2^	1606 ± 328 a	1628 ± 628 a	129 ± 45 b	0.001
*Sphingobacteriales* ^3^	6266 ± 861 a	5864 ± 209 a	8742 ± 792 b	0.032
Unclassified Bacteria	3738 ± 223 a	3775 ± 109 a	2069 ± 476 b	0.005
*Verrucomicrobia*	2578 ± 519 a	1845 ± 415 ab	1096 ± 86 b	0.024
*Verrucomicrobiales*	2125 ± 286 a	3836 ± 339 b	3576 ± 492 ab	0.024
Water				
*Caulobacterales* ^1^	395 ± 191 a	395 ± 106 a	1657 ± 157 b	<0.001
*Rhodobacterales* ^2^	708 ± 174 a	262 ± 106 a	24 ± 6 b	0.008
*Spartobacteria*	1295 ± 291 a	1641 ± 268 ab	2685 ± 315 b	0.021
*Sphingobacteriales* ^3^	4482 ± 477 a	4113 ± 551 a	6956 ± 527 b	0.007
*Sphingomonadales*	1020 ± 287 ab	2658 ± 869 a	414 ± 63 b	0.008
*Xanthomonadales*	1577 ± 819 ab	1053 ± 379 a	4399 ± 919 b	0.032

Numerical values represent average abundance within treatments ± standard error (*n* = 4). Superscript numbers indicate taxa that differed in both habitat types, and different lower-case letters represent significant differences in taxa across treatment groups.

**Figure 4:**
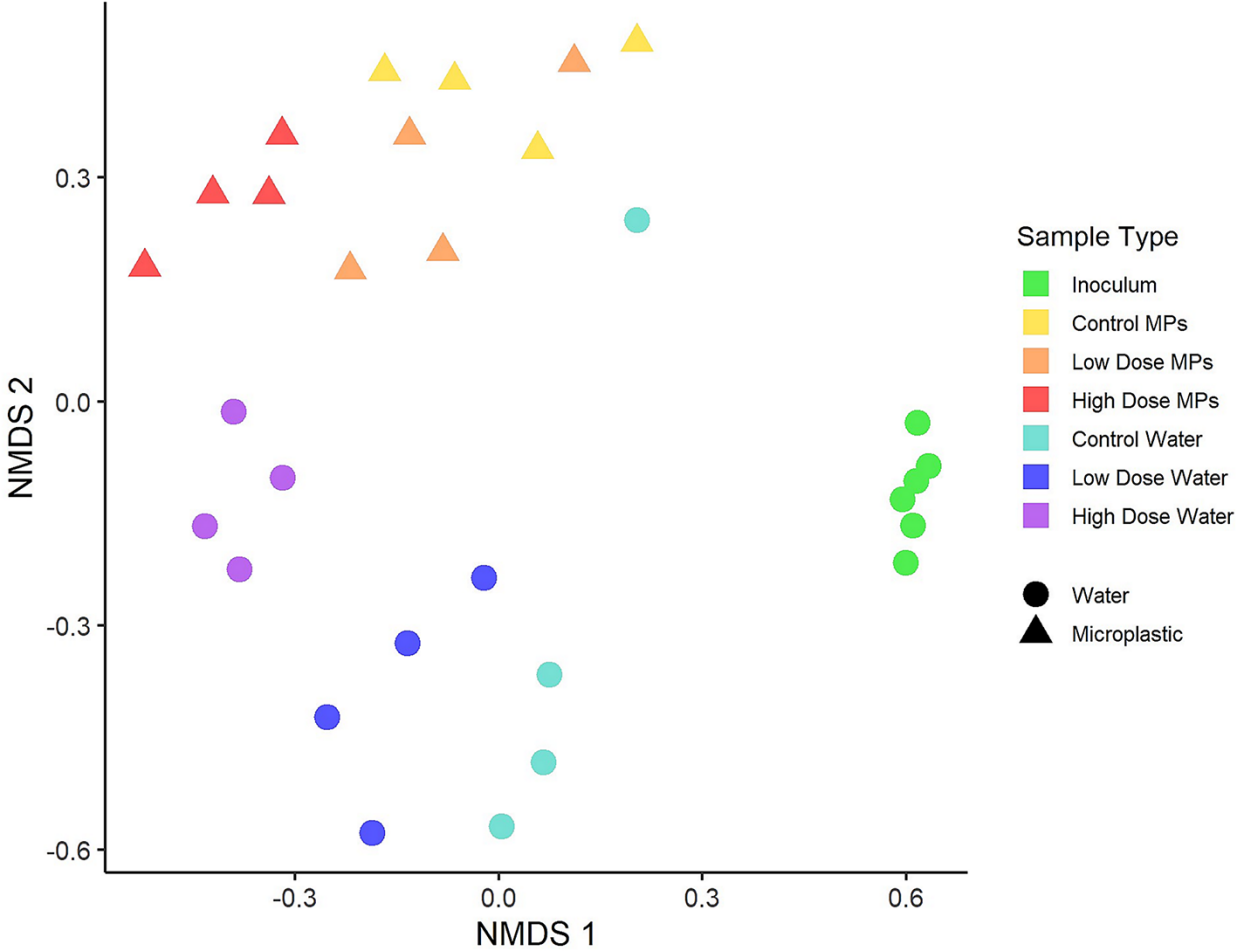
Non-metric multidimensional scaling (NMDS) ordination based on the Bray–Curtis dissimilarity scores of inoculum, MP, and water samples (stress = 0.238, R-squared = 0.802).

Broad shifts in the abundance of bacterial orders were corroborated by differences in beta diversity among treatments. Ordination based on antibiotic dosage and habitat indicated clear separation between communities on MPs and in water (Figure 4). Analysis of molecular variance (AMOVA) indicated significant differences between habitat types (inoculum, MP, or water; *F*_0.05, 2, 27_ = 17.569, *p* < 0.001). Within habitat types, high dose samples were significantly different than both low dose and untreated control samples (*p* < 0.05), but low dose and control samples did not significantly differ (Supplementary Table S10). Additionally, as antibiotic dosage increased, the beta diversity of MP and water samples became more similar, indicating that antibiotic contamination had a stronger impact on these microbial assemblages than habitat type.

### 3.3 Effects of antibiotics on ARG abundance

**Figure 5:**
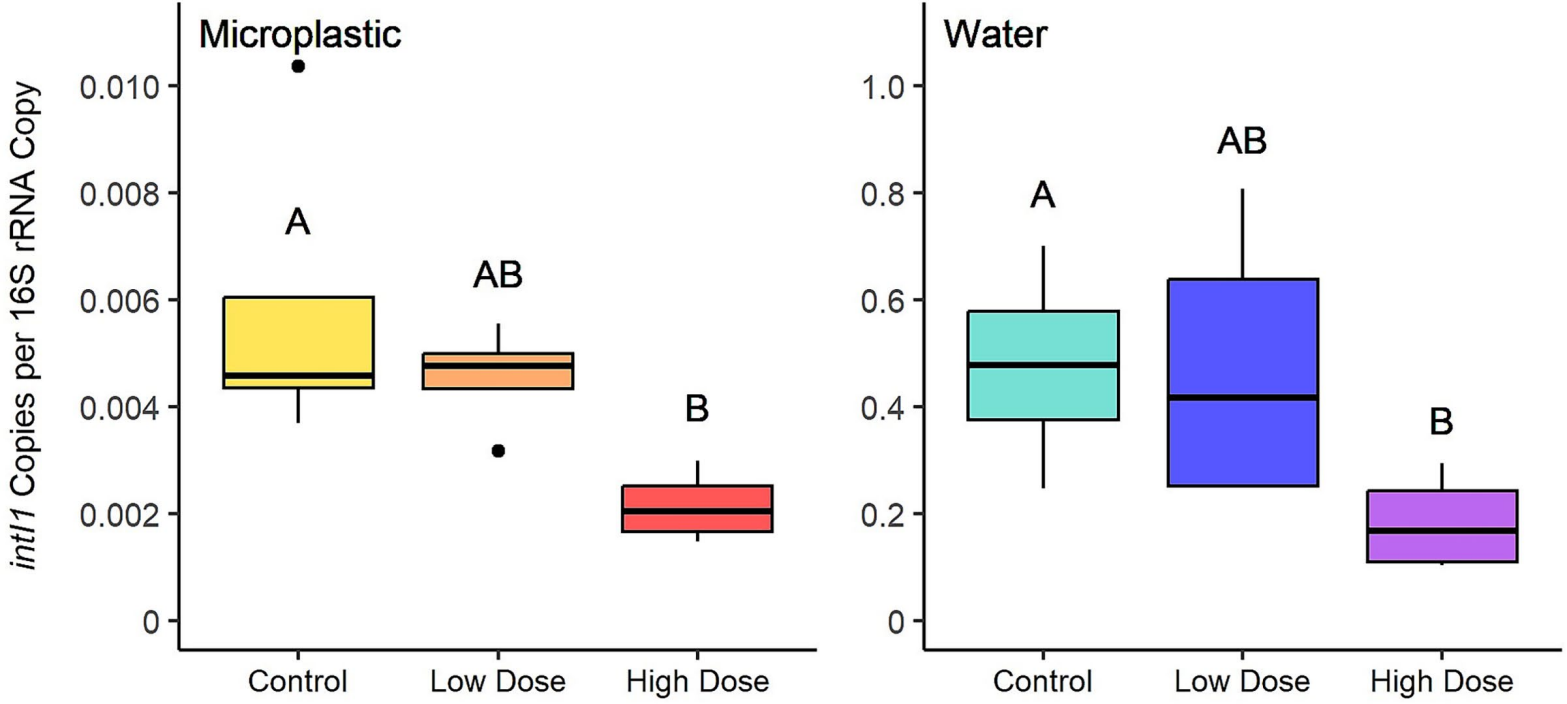
Abundance of the class 1 integrase gene *intI1* on MPs and in water (*n* = 4). Gene abundance was significantly lower on MPs compared to water (ANOVA; *p* < 0.001), and high dose samples had significantly lower *intI1* compared to controls (Tukey’s HSD; *p* < 0.05). Abundance did not significantly differ between high dose and low dose samples.

**Figure 6:**
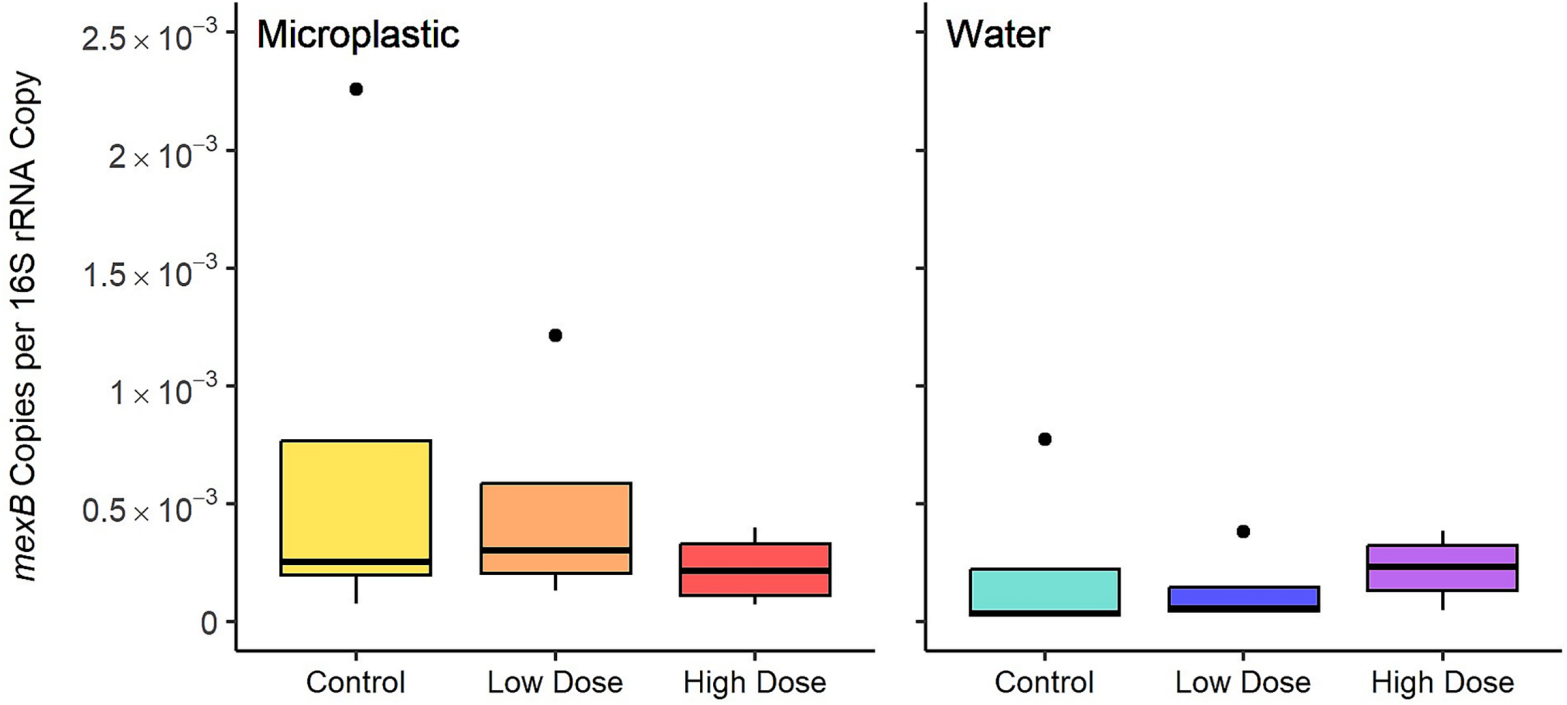
Abundance of the multidrug efflux pump gene *mexB* on MPs and in water (*n* = 4). MP and water samples did not significantly differ according to antibiotic dosage or habitat type.

**Figure 7:**
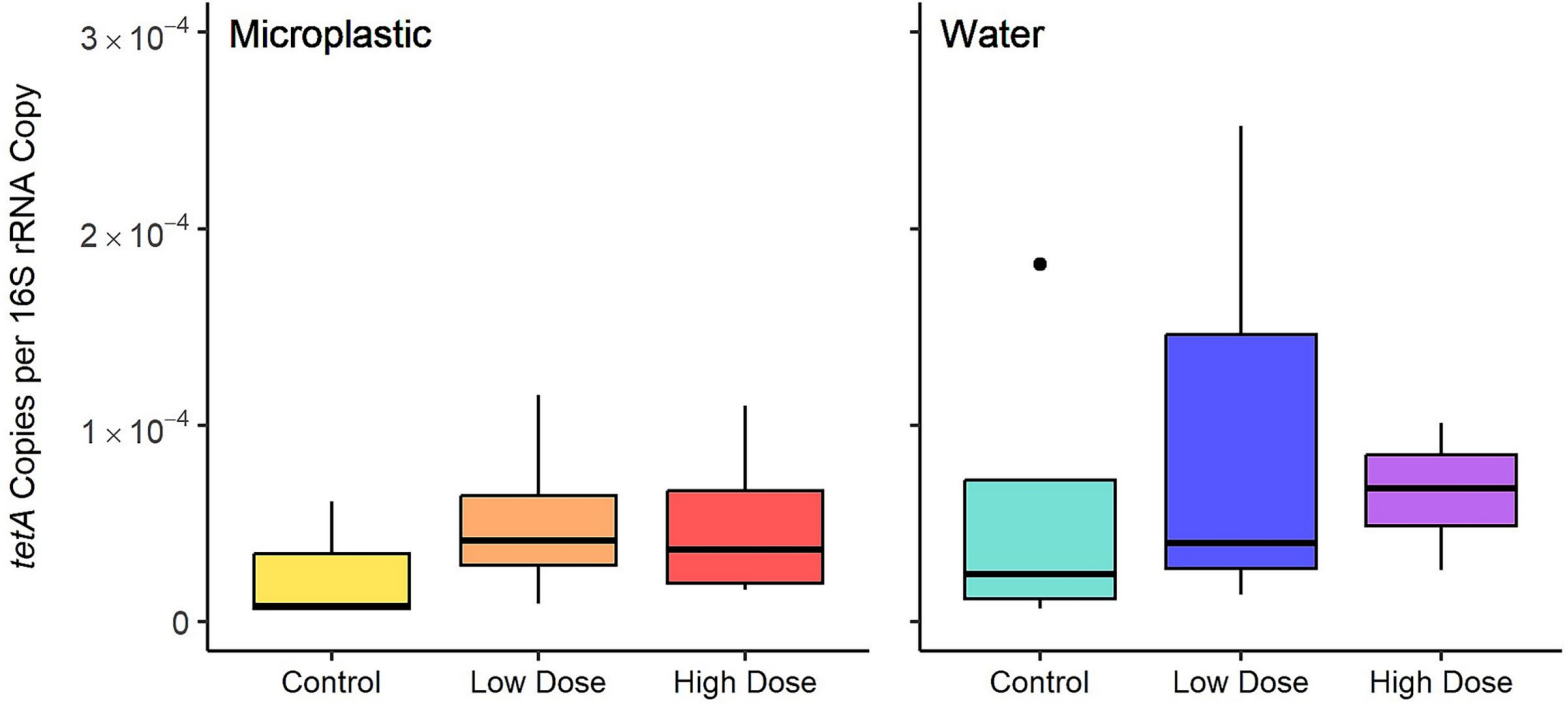
Abundance of the tetracycline efflux pump gene *tetA* on MPs and in water (*n* = 4). MP and water samples did not significantly differ according to antibiotic dosage or habitat type.

Three of the ten ARGs targeted in this study were detected via end-point PCR: the class 1 integrase gene *intI1*, the multidrug efflux pump *mexB*, and the tetracycline efflux pump *tetA*. Abundance of *intI1* was significantly greater in water than on MPs, and *intI1* was significantly less abundant among high dose MP and water samples compared to untreated control MP and water samples (Figure 5). In low dose samples, *intI1* abundance did not statistically differ from either control or high dose samples. Conversely, the efflux genes *mexB* and *tetA* were not abundant, particularly on MP surfaces, and neither varied according to antibiotic dosage or habitat type (Figures 6, 7).

## 4 Discussion

### 4.1 Adsorption of antibiotics to microplastics

Consistent with our predictions, several of the antibiotics added to the streams adsorbed to polyester MPs. Ciprofloxacin demonstrated the strongest adsorption on MP surfaces, reaching a concentration of 5.6 μg g^−1^ in the low dose treatment and 24.4 μg g^−1^ in the high dose treatment. Strong adsorption of ciprofloxacin to several MP polymers in freshwater has been reported previously (Li et al., 2018). Trimethoprim also adsorbed to MPs in both treatments, reaching 0.75 μg g^−1^ on MP surfaces in the high dose treatment. Previous reports show trimethoprim adsorbs to MP polymers in freshwater to a lesser extent than ciprofloxacin (Li et al., 2018), which agrees with the results of our study. Finally, sulfamethoxazole showed measurable adsorption to MPs only in the high dose treatment (47 ng g^−1^) and was below the adjusted detection limit (15 ng g^−1^) in the low dose and control streams, likely reflecting irreversible adsorption consistent with previous studies describing adsorption of sulfamethoxazole to multiple MP polymers (Guo et al., 2019).

Contrary to our expectations, amoxicillin, azithromycin, and nitrofurantoin were not detected on MP surfaces in any of our treatments. Previous studies have reported the adsorption of amoxicillin (Li et al., 2018; Wang et al., 2023) and azithromycin (González-Pleiter et al., 2021) to MPs, so we anticipated that adsorption would occur. However, multiple factors can influence adsorption, including particle characteristics (e.g., plastic polymer type and surface weathering) and environmental conditions (e.g., pH and ionic strength) (Li et al., 2018). The specific combination of these factors in our streams may have led to the adsorption of some antibiotics and not others. In addition, differences in antibiotic stability and persistence can also affect their adsorption to MP surfaces. For example, nitrofurantoin is susceptible to photolysis in surface water (Biošić et al., 2021), which could account for its absence on MP surfaces in our study.

### 4.2 Effects of antibiotics on bacterial communities

Exposure to ciprofloxacin, the most abundant adsorbed antibiotic in our study (Table 1), has been shown to alter the composition of bacterial biofilm communities colonizing cellulose substrates in freshwater in prior studies (Rosi et al., 2018). In the current study, both high and low dose streams were treated with sub-inhibitory antibiotic concentrations, and we observed a similar response. MP-attached and planktonic bacterial communities from high dose streams were distinct from the other treatments, whereas communities in low dose streams did not differ from controls. This was a consistent trend across multiple analyses that was most apparent in the ordination of bacterial communities, which showed clear grouping by habitat type and gradual shifts corresponding to increases in antibiotic dosage (Figure 2). Communities from MP and water samples treated with antibiotics converged along the same trajectory, mirroring findings from a related project in which MP fibers were exposed to the broad-spectrum antimicrobial compound triclosan (Krantz et al., 2026). In that study, bacterial communities populating MP surfaces and in the surrounding water converged based on triclosan exposure. These experiments suggest that even at sub-inhibitory concentrations, antibiotics can affect the composition of planktonic and MP-attached biofilm communities and highlight that the effects strengthen as concentrations increase.

The abundance of several bacterial orders detected in high dose streams differed from low dose and control streams (Figure 1 and Table 2). In particular, the order *Sphingobacteriales* increased by 40–50% on high dose MP samples compared to other treatments. This order of Gram-negative, non-spore-forming bacilli is a large group of metabolically diverse organisms (Figueiredo et al., 2022) that commonly colonize plastics in the environment. *Sphingobacteriales* has been identified as an indicator species for polyester contamination (McCall, 2023) and is a candidate group for biodegradation of plastics, including polyester and polystyrene (Qiu et al., 2025; Roager and Sonnenschein, 2019). *Sphingobacteriales* is also abundant in wastewater effluent (Drury et al., 2013) which can contain contaminants this group can degrade, including herbicides, antibiotics, and plastic leachates (Kampfer, 2010; Romera-Castillo et al., 2024; Wicaksono et al., 2021). A previous study demonstrated that exposure to ciprofloxacin, the most abundant antibiotic on MP surfaces (Table 1), selected for *Sphingobacteriales* in bacterial biofilm communities on organic substrates in a stream (Rosi et al., 2018). In the current study, this taxon was enriched in all high dose samples and was significantly more abundant on MPs than in water (Table 2; ANOVA; F_0.05, 1, 22_ = 6.363; *p* = 0.02). All streams received the same microbial inoculum and the same number of MP fibers, so the observed enrichment of *Sphingobacteriales* in the high dose streams was likely driven by elevated antibiotic concentrations.

*Caulobacterales* was the only other bacterial order among the 20 most abundant orders that significantly increased in abundance on MP fibers in the high dose streams; these taxa were also significantly more abundant in high dose water samples compared to low dose and untreated control samples. *Caulobaterales* are Gram-negative, rod-shaped, non-spore-forming alphaproteobacteria that have been previously observed to colonize polyester MPs in aquatic systems (McCall, 2023; Vaksmaa et al., 2021). Furthermore, this group is often reported to colonize samples impacted by elevated antibiotic concentrations, including ciprofloxacin (Hayes et al., 2025; Wicaksono et al., 2021). *Caulobacterales* taxa can harbor genes encoding putative hydrolytic dehalogenases capable of metabolizing ciprofloxacin and other halocarbons (Lavecchia et al., 2024), potentially explaining this observation. Both *Caulobacterales* and *Sphingobacteriales* include pathogenic taxa (Ryan and Pembroke, 2018; Wisplinghoff, 2017), so the enrichment of these orders by multiple antibiotics may have public health implications that could be compounded by MP transport through urban streams.

The order *Xanthomonadales* was more abundant on MPs compared to water samples (ANOVA: *F*_0.05, 1, 22_ = 31.71; *p* < 0.001), but it only increased in abundance in high dose water samples compared to other treatments. *Xanthomonadales* are Gram-negative, non-spore-forming, obligate aerobe gammaproteobacteria, and due to the prevalence of generalist hydrocarbon-degraders within this group (Gutierrez, 2017), *Xanthomonadales* taxa are consistently reported on MPs in diverse habitats (Miao et al., 2023; Xu et al., 2024). Furthermore, many *Xanthomonadales* taxa can sustain microbial community metabolism by performing glycolysis even under antibiotic stress (Shi et al., 2025), indicating a capacity for antibiotic resistance. *Xanthomonadales* includes several important phytopathogenic species (Naushad and Gupta, 2013) in addition to human pathogens that display multidrug resistance (Majumdar et al., 2022), so widespread contamination of the environment by MPs and antibiotics could increase the impact of this group on human health and agriculture.

While it is reasonable to suggest that the adsorbed antibiotics were the key driver of the observed differences in the MP-attached bacterial communities in the high dose streams, it is also possible that differences in these communities were caused by the antibiotics in the stream water changing the pool of bacteria available to colonize MP surfaces. The latter possibility is supported by the fact that the planktonic and MP-attached bacterial communities were more similar in the high dose treatment than in the control (Figure 4) and is further supported by similar patterns in the abundance of several bacterial orders observed in the water and on MP surfaces (Table 2). For example, *Caulobacterales* and *Sphingobacteriales* were more abundant in both the high dose water and MP samples. Similar responses to the high dose antibiotic treatment that were observed in the planktonic and surface-attached bacterial communities were noteworthy because these different bacterial lifestyles are associated with different responses to antibiotics, with bacteria in biofilms being significantly more resistant to antibiotics than planktonic cells (Uruén et al., 2021). However, there were also several bacterial orders whose responses to the antibiotic treatment were distinct in the water as compared to the MPs (Table 2). For example, *Xanthomonadales* and *Spartobacteria* were more abundant in the high dose water but were not more abundant on the high dose MPs, and *Sphingomonadales* were less abundant in the high dose water but did not differ in abundance on the high dose MPs.

Any attempt to definitively identify the specific location of the antibiotic-bacterial interactions will be confounded by the dynamic adsorption and desorption of the antibiotics from MP surfaces and the interactions between the planktonic and surface attached microbial communities. For example, in a previous experiment by our group an antimicrobial compound was adsorbed to the surface of MP fibers prior to the addition of these fibers to a freshwater microcosm, yet both the MP-attached and planktonic microbial communities in the microcosms were impacted by the adsorbed antimicrobial compound (Krantz et al., 2026). In addition, the fact that we only measured the MP-adsorbed antibiotics and not the dissolved antibiotics limits our insight into the possible impacts of the dissolved antibiotics. Based on data collected in the current study it seems most likely that both the adsorbed and dissolved antibiotics played a role in shaping the MP-attached bacterial communities.

The high dose treatment in the current study is representative of antibiotic concentrations detected in untreated wastewater, and the low dose treatment is representative of antibiotic concentrations in urban streams. Consequently, these results suggest that antibiotic interactions with MPs and bacterial communities will likely be strongest in wastewater, and that adequate wastewater treatment is necessary to mitigate the strongest effects of antibiotic and MP contamination on natural microbial communities and processes. However, damaged wastewater infrastructure, combined sewer overflows, or the lack of sufficient treatment facilities can all lead to the direct emission of concentrated antibiotics and MPs into the environment (Di Nunno et al., 2021; Woodward et al., 2021). When treated wastewater effluent is released into the environment, our data suggest that dilution of the antibiotics and MPs may reduce their interaction, and thus the potential for antibiotics to structure MP biofilms *in situ*. Simultaneously, WWTP effluent accounts for greater than 50% of the flow of more than 900 rivers in the United States (Rice and Westerhoff, 2017), and when wastewater makes a major contribution to the flow of a receiving stream, antibiotic interactions with MPs and bacterial communities may persist.

The antibiotic treatments implemented in this study were composed of a mixture of eight antibiotics added at two environmentally relevant concentrations. We designed this treatment to reflect the complex mixtures and varied concentrations of antibiotics that occur in wastewater and aquatic environments. We recognize that adding a mixture of antibiotics creates the potential for synergistic and/or antagonistic effects, which could not be isolated with this study design. Future experiments could include antibiotics both singly and in mixtures to enable assessment of synergistic and antagonistic effects, although also substantially increasing the complexity of the experimental design.

### 4.3 Effects of antibiotics on ARG abundance

Two ARGs were detected in samples from this study: *mexB* and *tetA*. Failure to detect the other seven ARGs, even in the inoculum, indicates that they were not abundant in the communities sourced from the Chicago River. Contrary to our predictions, the two ARGs that were detected did not vary in abundance with antibiotic dosage (Figures 6, 7). The ARG *mexB* encodes a multidrug efflux pump that confers resistance to beta-lactam antibiotics, including amoxicillin, and fluoroquinolones, including ciprofloxacin (Dreier and Ruggerone, 2015). Antibiotic treatments in the current study contained both amoxicillin and ciprofloxacin, and ciprofloxacin in particular strongly adsorbed to the MPs; nonetheless, these antibiotics did not select for increased abundance of *mexB*. The ARG *tetA* encodes an efflux pump that confers resistance to tetracyclines (Chopra and Roberts, 2001) including doxycycline, which was included in our antibiotic treatments, but we observed no correlation between antibiotic dosage and *tetA* abundance. That antibiotic exposure did not affect ARG abundance in our study suggests either that the antibiotic doses we implemented were not high enough to impact ARG selection, or that selection may require a longer exposure period than the short duration of our study (18 days), which may not have provided adequate time for their horizontal transfer within MP biofilm communities. Additionally, it is possible that the antibiotic treatments may have rapidly selected for naturally resistant taxa, e.g., *Caulobacterales* and *Sphingobacteriales*, which are not reliant upon ARGs. Finally, it is possible that the antibiotic treatment may have affected the expression of these ARGs without affecting their abundance. Future work could incorporate reverse transcriptase-qPCR or metatranscriptomics to assess ARG expression.

Although the observed decrease in *intI1* in the high dose MP and water treatments contradicted our predictions, it also indicated an impact of antibiotics on the resistance profiles of both planktonic and MP-associated bacterial communities (Figure 5). The class 1 integrase gene is a component of an operon that enables bacteria to incorporate environmental DNA, often containing ARGs, into the bacterial genome (Hall and Collis, 1995; Stokes and Hall, 1989), and as such it can be used as an indicator of anthropogenic impacts on environmental microbial communities (Gillings et al., 2015). The decreases in *intI1* abundance that we observed in response to increasing antibiotic concentrations therefore indicate an overall decrease in the capacity of bacteria at high concentrations to horizontally acquire ARGs from the environment, contrary to our initial hypothesis. The decrease in *intI1* abundance may be linked to selection of naturally resistant taxa (Dantas et al., 2008): the antibiotic cocktails contained compounds with diverse functionality, limiting the growth of organisms without inherent multidrug resistance in the short term. Such taxa might not rely on *intI1* to access ARGs in the environment, leading to a relative decrease in its abundance in high dose mesocosms.

## 5 Conclusion

Our findings reiterate the significant impact of increased antibiotic concentrations on community composition and the differences between bacterial communities colonizing MPs and the surrounding water. Elevated concentrations of MPs and antibiotics selected for distinct communities in both habitats, including several pathogenic and plastic-degrading taxa relevant to human health and commerce. Paired with the propensity for MP particles to transport unique MP-associated bacterial assemblages through aquatic systems, these observations support the concern for ubiquitous MP contamination in wastewater and the environment. Quantification of the class 1 integrase gene *intI1* in our samples showed additional impacts of antibiotics on both surface-attached and planktonic bacterial communities. In contrast to our initial predictions, we observed a significant decrease in the abundance of *intI1* in MP and water samples exposed to high concentrations of antibiotics and we did not measure significant differences in the abundance of the antibiotic efflux pump genes *mexB* and *tetA.* Future work should include metagenomic analysis to detect additional ARGs, possibly painting a more complete picture of the effects of elevated antibiotic concentrations on ARG abundance. Because wastewater is a point source of MPs and antibiotics to the environment, and MP particles transport unique MP-associated bacterial assemblages through aquatic ecosystems, our data suggested MP contamination in wastewater can have far-reaching environmental impacts.

## Data Availability

All sequence data analyzed in this paper can be downloaded from the NCBI Sequence Read Archive (SRA) with accession number PRJNA1448104.
