# Supplementary material for: Antibiotics adsorb to polyester microplastic fibers and alter bacterial communities in stream mesocosms
Source: Front Microbiol. 2026 Jul 23;17:1847208. doi: 10.3389/fmicb.2026.1847208 (PMC13442399; doi:10.3389/fmicb.2026.1847208)
Supplement: Supplementary file 1 [file Data_Sheet_1.pdf]

Antibiotics Adsorb to Polyester Microplastic Fibers and Alter Bacterial Communities in Stream Mesocosms

Ryan T. Krantz [1], Sathaporn Onanong [2], Daniel D. Snow [2], Timothy J. Hoellein [1], and John J. Kelly [1,*]

[1] Department of Biology, Loyola University Chicago, Chicago, IL, USA

[2] Water Sciences Laboratory, University of Nebraska-Lincoln, Lincoln, NE, USA

Supplementary Material

* Correspondence:

John J. Kelly

phone: 773.508.3681

email: jkelly7@luc.edu

Antibiotic	Influent	Effluent	Stream
Amoxicillin	280 [a]	190 [b]	68 [c]
Azithromycin	6842 [d]	6170 [d]	1600 [e]
Ciprofloxacin	6315 [d]	3403 [b]	383 [f]
Doxycycline	10000 [g]	10900 [g]	82 [h]
Gentamicin	1600 [i]	500 [i]	–
Nitrofurantoin	9300 [j]	–	–
Sulfamethoxazole	5787 [d]	12848 [b]	100 [f]
Trimethoprim	1779 [d]	5316 [b]	1069 [k]
**Average**	**5238**	**5618**	**550**

Table S1. Maximum reported concentrations (ng L^{-1}) of antibiotics in untreated wastewater influent (sewage), treated wastewater effluent, and in urban streams. Antibiotic dosages in this study are based on averages calculated from these reported values. [a] Watkinson et al., 2007; [b] Dinh et al., 2017; [c] Q. T. Dinh et al., 2011; [d] Majeed et al., 2021; [e] Bielen et al., 2017; [f] Booth et al., 2020; [g] Karthikeyan & Meyer, 2006; [h] Deng et al., 2016; [i] Tahrani et al., 2015; [j] Hricová et al., 2021; [k] Xu et al., 2015.

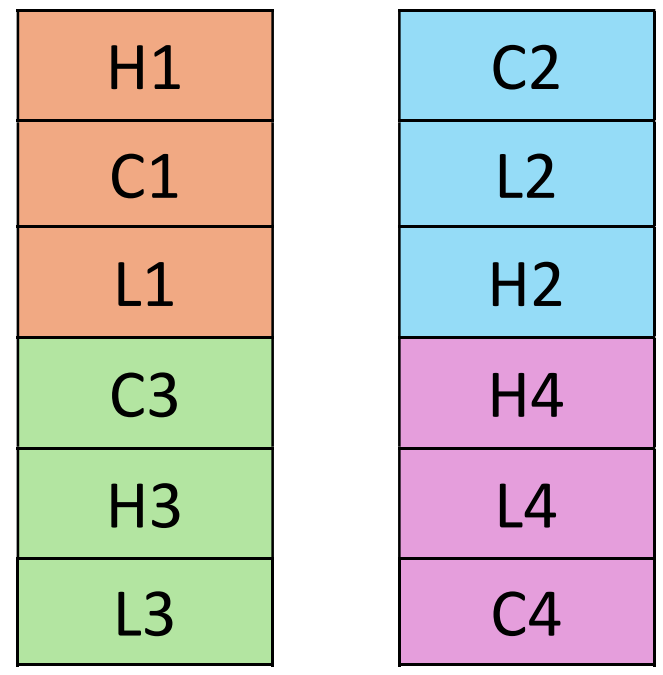

Figure S1: Artificial stream mesocosms were organized into four quadrants, each containing one replicate per experimental dose. Within quadrants, treatments were interspersed to account for effects of stream position within the greenhouse laboratory. C = control (no added antimicrobials), L = low dose, H = high dose. Numbers indicate replicate mesocosms.

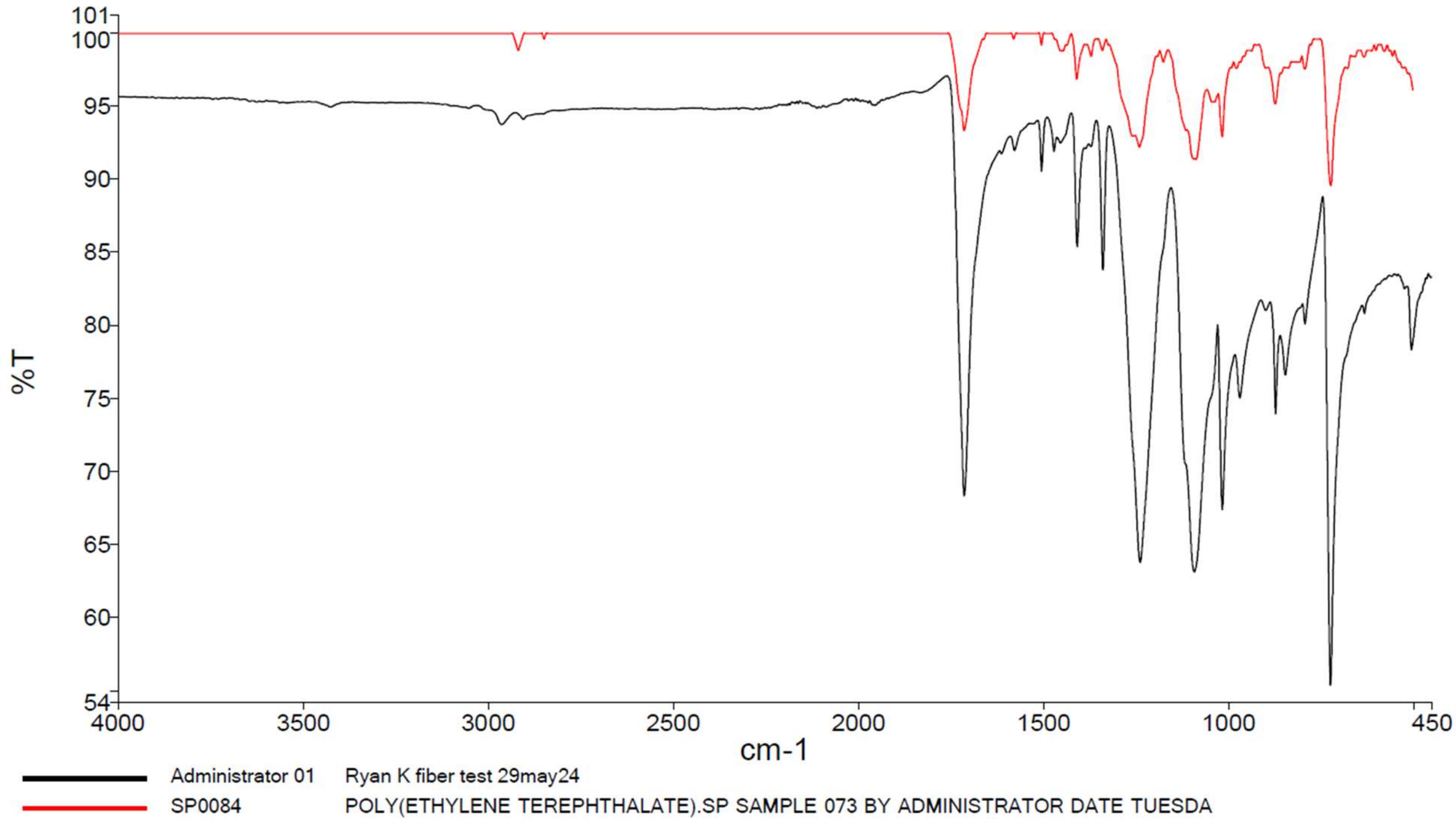

Figure S2: Micro-Fourier transform infrared (FT-IR) spectra matching yarn marketed as 100% recycled polyester to poly(ethylene terephthalate) with a correlation of 0.886.

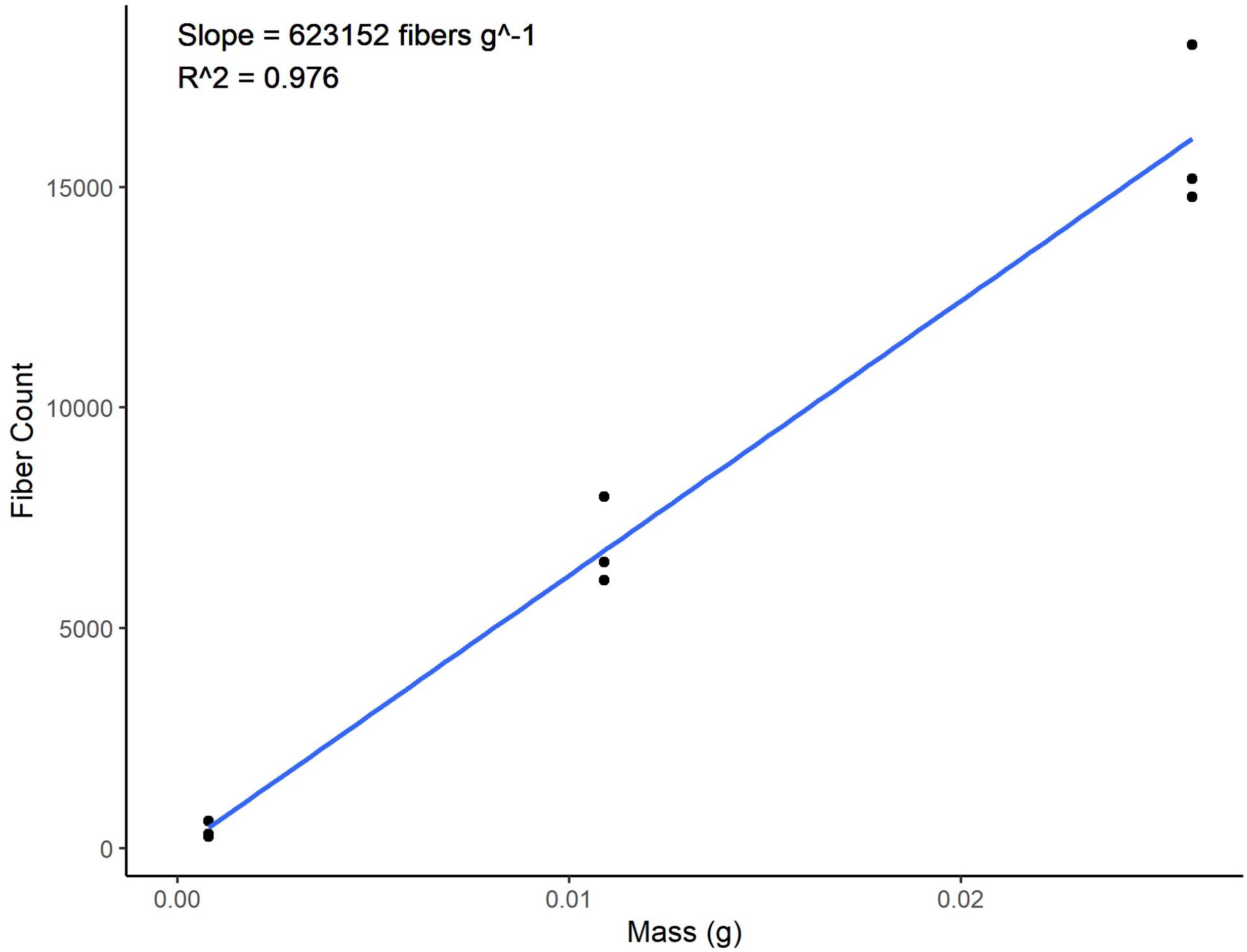

Figure S3: Fiber count × mass regression used to calculate number of fibers per gram of polyester yarn.

Antibiotic	Cat	Lot
Amoxicillin trihydrate	J61290.14	U17J549
Azithromycin dihydrate	J66740.06	U24J019
Ciprofloxacin, 98%	J61317.14	P15K025
Doxycycline hyclate	J60579.14	M25K031
Gentamicin sulfate, 600 IU / mg	J62834.09	P08K040
Nitrofurantoin, 98%	B24079.14	U09J012
Sulfamethoxazole, 98%	J66565	W24F032
Trimethoprim	J63053.06	X04K023

Table S2: Antibiotics used in this study. All compounds were purchased as powders from Thermo Fisher Scientific (Ward Hill, MA).

**Water Parameter**	**Read 1**	**Read 2**	**Read 3**	**Average (± SE)**
Temp (°C)	16.3	16.3	16.3	**16.3 (0)**
Pressure (mmHg)	737.2	737.0	737.0	**737.1 (0.1)**
Dissolved Oxygen (% L)	69.3	73.4	69.5	**70.7 (1.3)**
Dissolved Oxygen (mg L^{-1})	6.58	6.96	6.59	**6.71 (0.13)**
Conductivity (µS cm^{-1})	656	655	656	**656 (0)**
Total Dissolved Solids (mg L^{-1})	426	426	426	**426 (0)**
Salinity (ppt)	0.32	0.32	0.32	**0.32 (0)**
pH	7.95	7.92	7.91	**7.93 (0.01)**
pH mV	-75.8	-74.4	-73.3	**-74.5 (0.7)**
Oxidation-Reduction Potential (mV)	97.6	99.3	99.6	**98.8 (0.6)**
Chlorophyll (RFU)	2.46	2.66	2.57	**2.56 (0.06)**
Phycocyanin (RFU)	-0.33	0.18	-0.27	**-0.14 (0.16)**
Depth (m)	0.351	0.298	0.284	**0.311 (0.02)**

Table S3: Parameters of water from the North Branch of the Chicago River at Harms Woods, Glenview, IL, as measured by a YSI ProDSS multiparameter water quality meter (Yellow Springs, OH).

Ion	Sample 1	Sample 2	Sample 3	Sample 4	Average (± SE)
Sodium	43.524	41.879	47.128	28.481	**40.253 (4.074)**
Potassium	4.427	3.973	4.484	2.623	**3.877 (0.433)**
Calcium	27.479	24.896	28.693	18.689	**24.939 (2.229)**
Magnesium	9.721	9.874	10.956	9.271	**9.956 (0.357)**
Chloride	66.022	59.785	68.519	40.963	**58.822 (6.230)**
Bromide	0.096	0.108	0.116	0.075	**0.099 (0.009)**
Nitrate	13.781	12.051	13.962	8.617	**12.103 (1.239)**
Phosphate	0.220	0.217	0.256	0.012	**0.176 (0.055)**
Sulfate	28.317	27.895	31.394	21.349	**27.239 (2.112)**

Table S4: Average concentrations (mg L^{-1}) of ions detected in subsamples of river water inoculum. Ammonium, fluoride, and nitrite concentrations were below the limit of detection.

Antibiotic Analysis Instrument Conditions and Validation

Details for modified EPA 1694 group 1 human use subgroup compounds measured in microplastic samples using the Xevo TQ-S Micro UPLC/MS/MS (Waters Corporation, Milford, MA USA) with an Acquity H-PLUS solvent management system and Uni-Spray™ ionization source. Two compounds, amoxicillin and nitrofurantoin, were added to this method after establishing suitable instrumental conditions and transitions. Chromatographic separation and instrumental analysis were performed by using Acquity Premier BEH C_{18} VanGuard Fit reverse phase UPLC column (50 mm x 2.1 mm x 1.7 μm) at a flow rate of 0.6 mL min^{-1} and temperature of 40°C.

Source conditions are listed in Table S5. Mobile phase solvents were: A) 0.3% (v/v) formic acid with 0.1% (w/v) ammonium formate in water and B) 50% methanol/50% acetonitrile. Flow rate was 0.6 mL/min. Initial mobile phase conditions were 100:0 A/B, gradually increase to 70:30 A/B at 1 min and 30:70 A/B at 5 min, hold for 0.5 min and switch to 0:100 A/B, hold for 3.5 min before switching back to the original condition of 100:0 A/B at 9 min. Total run time was 12 min per sample. Injection volume was 2 μL.

Ionization Mode	Positive
Impactor Voltage (kV)	1.5
Source Temperature (°C)	150
Desolvation Temp (°C)	600
Desolvation Gas Flow (L/Hr)	1000
Cone Gas Flow (L/Hr)	1
LM Resolution 1	4.7
HM Resolution 1	15.1
Ion Energy 1	-0.5
Entrance	1
Exit	1
LM Resolution 2	7.6
HM Resolution 2	15.2
Ion Energy 2	0.5
Ion Guide 2 Offset (V)	0.5
Detector Gain	1

Table S5. Antibiotic analysis source conditions.

Compound	Parent-Product Ion (m/z) transitions	Cone (V)	Collision (V)	Retention Time (min)
Acetaminophen-(acetyl-13C2,15N) (IS)	155.108>92.949<br>155.108>44.939	36	26<br>24	2.17
Ciprofloxacin-(carboxyl-13C3, quinoline-15N) (IS)	336.068>318<br>336.068>248.105	54	20<br>24	2.76
Caffeine-(trimethyl-13C3) (IS)	198.074>42.926<br>198.074>140.034	46	46<br>28	2.58
Trimetroprim-(pyrimidine-13C3) (IS)	294.14>126.042<br>294.14>264.043	64	30<br>32	2.67
Carbamazepine-13C6 (IS)	243.11>171.049<br>243.11>184.905	42	50<br>44	4.91
Sulfamethazine-(phenyl-13C6) (IS)	285.067>97.953<br>285.067>123.971	44	44<br>34	2.73
Sulfamethoxazole-13C6 (IS)	260.134>98.071<br>260.134>162	16	38<br>20	3.00
Thiabendazole-13C6 (IS)	208.062>180.982<br>208.062>137.003	54	22<br>32	2.66
Erythromycin-(N-methyl-13C, D3) (IS)	738.398>82.986<br>738.398>162.130	16	70<br>48	5.07
Cotinine-(methyl-d3) (IS)	180.134>80<br>180.134>100.949	52	32<br>30	1.14
Fluoxetine-d6 (IS)	316.170>43.934<br>316.170>154.134	34	20<br>10	5.33
Atrazine-13C3 (Sur)	219.117>97.966<br>219.117>105.903	46	32<br>40	5.00
Des-ethyl atrazine-13C3 (Sur)	191>149<br>191>82	30	20<br>20	3.07
Orphenadrine (Sur)	270.093>181.129<br>270.093>165.258	12	16<br>62	5.03
Oleandomycin (Sur)	688.381>158.159<br>688.381>544.271	42	30<br>18	4.86
1,7-Dimethylxanthine	181.2>124<br>181.2>96	40	25<br>35	2.39
Acetaminophen	152.064>92.828<br>152.064>64.979	50	22<br>28	2.17
Amoxicillin MeOH	398.276>114.080<br>398.276>349.159	34	44<br>14	2.70
Ampicillin	351.846>265.112<br>351.846>43.964	60	24<br>38	2.81
Azithromycin	749.438>158.159<br>749.438>83.048	62	38<br>60	5.44
Caffeine	195.094>138.027<br>195.094>41.948	60	18<br>34	2.58
Carbamazepine	237.11>178.953<br>237.11>165.094	36	34<br>38	4.91

Ciprofloxacin	332.117>314<br>332.117>231.076	54	15<br>36	2.76
Clarithromycin	748.344>158.099<br>748.344>83.049	44	28<br>50	5.44
Codeine	300.137>58.014<br>300.137>215.084	68	30<br>24	2.47
Cotinine	177.124>80.014<br>177.124>97.973	40	20<br>20	1.15
Danofloxacin	358.157>340<br>358.157>82.073	58	15<br>48	2.81
Dehydronifedipine	345.077>284.098<br>345.077>267.972	64	26<br>26	5.22
Digoxigenin	391.159>355.171<br>391.159>337.218	46	10<br>14	3.29
Digoxin	781.344>97.029<br>781.344>651.297	74	28<br>10	5.19
Diltiazem	415.064>178.006<br>415.064>108.938	56	24<br>60	4.98
Diphenhydramine	256.19>167.024<br>256.19>152.001	34	14<br>48	4.69
Enrofloxacin	360.3>342.3<br>360.3>316.3	56	20<br>20	2.85
Erythromycin	734.334>158.094<br>734.334>83.047	54	28<br>56	5.07
Flumequine	262.09>202.003<br>262.09>126.009	30	30<br>48	4.84
Fluoxetine	310.17>43.963<br>310.17>148.103	38	10<br>6	5.34
Lincomycin	407.189>126.104<br>407.189>41.821	46	28<br>70	2.59
Lomefloxacin	352.127>265.081<br>352.127>43.962	68	22<br>30	2.82
Miconazole	416.97>158.505<br>416.97>161.426	80	28<br>26	6.07
Nitrofurantoin	239.253>95.011<br>239.253>121.959	50	14<br>18	2.68
Norfloxacin	320.107>302<br>320.107>233.106	62	15<br>22	2.74
Norgestimate	370.149>124.029<br>370.149>91.052	84	30<br>46	6.30
Ofloxacin	362.083>318<br>362.083>261.056	66	15<br>26	2.73
Ormetoprim	275.108>123.054<br>275.108>81.037	62	24<br>40	2.76

Oxacillin (MeOH)	434.068>160.006<br>434.068>144.007	46	14<br>32	5.07
Penicillin G (MeOH)	367.1319>160.012<br>367.1319>91.074	2	14<br>56	4.58
Penicillin V (MeOH)	383.004>160.015<br>383.004>114.033	36	14<br>38	4.96
Phenazone	189.132>55.999<br>189.132>77.014	50	28<br>36	2.85
Roxithromycin	837.38>158.096<br>837.38>116.021	62	38<br>46	5.48
Sarafloxacin	386.137>299.035<br>386.137>348.068	52	24<br>30	3.01
Sucralose (Na)	418.904>220.993<br>418.904>238.876	52	18<br>18	2.82
Sulfachloropyridazine	285.1>156<br>285.1>92	32	15<br>28	2.91
Sulfadiazine	251.057>91.967<br>251.057>155.968	44	26<br>12	2.32
Sulfadimethoxine	311.107>91.962<br>311.107>156.023	58	32<br>18	3.68
Sulfamerazine	265.087>91.964<br>265.087>155.962	50	26<br>16	2.56
Sulfamethazine	279.107>91.973<br>279.107>186.033	36	30<br>16	2.73
Sulfamethizole	271.043>91.973<br>271.043>155.981	38	28<br>12	2.74
Sulfamethoxazole	254.057>92.032<br>254.057>155.973	14	24<br>14	3.00
Sulfanilamide	173.104>92<br>173.104>156	38	20<br>10	0.61
Sulfathiazole	255.969>155.987<br>255.969>91.975	34	14<br>26	2.47
Thiabendazole	202.026>175.017<br>202.026>131.052	36	22<br>30	2.66
Trimethoprim	291.098>123.053<br>291.098>230.041	68	22<br>22	2.67

Table S6. Multiple reaction monitoring (MRM) transitions, cone voltage, collision energies and representative retention times for internal standards (IS), surrogates and target compounds in the modified EPA 1694 Group 1 instrumental protocol. Transitions for additional compounds identified using MassLynx IntelliStart software.

Compound	IDL (pg)	MDL (ng g^{-1})	AVG % REC	REL % S
1,7-Dimethylxanthine	8.74	14.39	23.96	40.05
Acetaminophen	0.41	2.66	66.87	26.51
Amoxicillin MeOH	0.80	1.47	23.08	42.37
Ampicillin	0.88	11.89	13.06	57.90
Azithromycin	11.10	1.43	67.49	14.18
Caffeine	0.62	12.38	73.30	11.27
Carbamazepine	14.12	0.63	69.61	6.02
Ciprofloxacin	8.85	21.16	116.80	11.53
Clarithromycin	0.29	1.23	64.64	12.70
Clinafloxacin	1.47	0.65	9.98	43.47
Codeine	0.32	2.03	61.83	21.94
Cotinine	0.06	3.05	37.41	54.46
Danofloxacin	4.55	1.05	24.14	29.03
Dehydronifedipine	0.85	1.77	91.08	12.95
Digoxigenin	1.05	0.88	91.99	6.10
Digoxin	10.37	14.60	59.64	16.33
Diltiazem	9.27	28.24	105.74	17.81
Diphenhydramine	0.80	1.53	27.50	37.00
Enrofloxacin	6.75	0.70	18.74	23.75
Erythromycin	0.11	0.19	6.77	18.63
Flumequine	0.71	0.86	74.29	7.71
Fluoxetine	1.80	0.09	1.09	54.94
Lincomycin	1.26	3.24	38.61	55.95
Lomefloxacin	2.67	1.53	27.81	36.79
Miconazole	0.52	0.06	0.58	68.91
Nitrofurantoin	4.68	6.94	37.36	11.82
Norfloxacin	0.67	0.19	4.92	25.62
Norgestimate	3.14	0.22	8.50	17.63
Ofloxacin	0.83	1.57	24.47	42.74
Ormetoprim	14.20	1.26	58.05	14.54
Oxacillin MeOH	10.77	4.52	19.90	15.15
Penicillin G MeOH	111.96	2.97	12.49	15.88
Penicillin V MeOH	5.41	3.07	14.66	13.96
Phenazone	1.64	2.10	99.51	14.06
Roxithromycin	0.96	2.71	93.65	19.33
Sarafloxacin	2.22	0.29	6.33	30.10

Sucralose Na	18.10	7.55	24.40	20.64
Sulfachloropyridazine	1.27	0.81	60.73	8.90
Sulfadiazine	0.70	1.96	100.66	12.96
Sulfadimethoxine	0.48	0.75	39.00	12.89
Sulfamerazine	1.43	1.62	111.14	9.71
Sulfamethazine	1.02	2.23	123.03	12.11
Sulfamethizole	0.48	1.61	68.64	15.60
Sulfamethoxazole	1.71	1.03	84.90	8.13
Sulfanilamide	15.41	19.93	39.06	34.03
Sulfathiazole	0.14	0.82	75.76	6.88
Thiabendazole	0.35	0.94	75.24	8.38
Trimethoprim	0.80	0.65	69.76	5.94

Table S7. Instrument detection limits (IDL) in picograms on-column estimated as 3x standard deviation of the lowest standard for compounds measured using the modified EPA Method 1694. Method detection limits (MDL) determined from the variability in replicate samples (n = 8) of 1-gram pre-cleaned polyethylene powder (Aldrich #434272-100G, ultra-high molecular weight, 40 – 48 μm particle size) fortified at 5 and 50 ng g^{-1} using a spike containing EPA 1694 Group 1 analytes, plus amoxicillin and nitrofurantoin.

Gene	Corresponding Antibiotic(s)	Positive DNA Template	Function	PCR T_a (°C)	qPCR T_a (°C)	Forward Primer	Reverse Primer	Citation
abeM	Ciprofloxacin Gentamicin Trimethoprim	*A. baumannii*	Multidrug efflux	52	55	TGC CAA TTG GTT TAG CTG TG	TAC TTG GTG TGC GGC AAT AA	Fernando et al., 2014
ampC	Amoxicillin	*E. cloacae*	Inactivation	59	59	TGG CGT ATC GGG TCA ATG T	CTC CAC GGG CCA GTT GAG	Miossec et al., 2013
ermA	Azithromycin Ciprofloxacin	*S. aureus*	Target alteration	46	46	TCT AAA AAG CAT GTA AAA GAA	CTT CGA TAG TTT ATT AAT ATT AGT	Sutcliffe et al., 1996
intI1	Mobile genetic element	Plasmid containing cloned amplicon [1]	Class 1 integrase	54.5	54	GCC TTG ATG TTA CCC GAG AG	GAT CGG TCG AAT GCG TGT	Barraud et al., 2010
lmrS	Azithromycin Ciprofloxacin Trimethoprim	*S. aureus*	Multidrug efflux	53	55	TGC GAT GGC GAT GTA GAT AAA	CTC ACA TGG CAC GGC TAT TA	Silva et al., 2023
mexB	Amoxicillin	*P. aeruginosa* (PAO1)	Multidrug efflux	55	55	GTG TTC GGC TCG CAG TAC TC	AAC CGT CGG GAT TGA CCT TG	Yoneda et al., 2005
oqxB	Nitrofurantoin	*K. pneumoniae*	Multidrug efflux	54	60	GGT ATC GCT AAC CGA GCT ATT C	TAC GCC CGA ACT GGT TAA AG	Hussein et al., 2023
sul1	Sulfamethoxazole	Environmental sample	Target replacement	60	60	CCG TTG GCC TTC CTG TAA AG	TTG CCG ATC GCG TGA AGT	Heuer & Smalla, 2007
tetA	Tetracyclines	Plasmid containing cloned amplicon [1]	Efflux	57	59	CTC ACC AGC CTG ACC TCG AT	CAC GTT GTT ATA GAA GCC GCA TAG	Looft et al., 2012
tetM	Doxycycline	*S. aureus*	Target protection	54	54	TAA TAT TGG AGT TTT AGC TCA TGT TGA TG	CCT CTC TGA CGT TCT AAA AGC GTA TTA T	Looft et al., 2012

Table S8: Primer and thermocycling information for genes targeted in this study. [1] Prepared according to methods detailed in Krantz et al., 2026.

Target Gene	Standard Template	R^2	Efficiency
16S rRNA	*P. aeruginosa* (PAO1)	0.976	100.210
intI1	Plasmid containing cloned amplicon	0.991	96.603
mexB	*P. aeruginosa* (PAO1)	0.994	77.731
tetA	Plasmid containing cloned amplicon	0.999	94.518

Table S9: Characteristics of qPCR standard curves. Standards were comprised of serially-diluted positive DNA template from 10^8 – 10^2 gene copies.

Microplastic		Low Dose	High Dose
Control	F_S	1.234	3.341
	p-value	0.199	**0.028**
High Dose	F_S	2.588	
	p-value	**0.037**	
**Water**			
Control	F_S	1.366	3.608
	p-value	0.109	**0.037**
High Dose	F_S	3.89	
	p-value	**0.033**	

Table S10: Analysis of molecular variance (AMOVA) based on Bray-Curtis dissimilarity scores of communities on MPs and in water, according to antibiotic dosage. High dose samples differed from low dose and control samples, both on MPs and in water. Differences among samples grouped by habitat type were also significant (all $p < 0.001$).

Literature Cited

Barraud, O., Baclet, M. C., Denis, F., & Ploy, M. C. (2010). Quantitative multiplex real-time PCR for detecting class 1, 2 and 3 integrons. *Journal of Antimicrobial Chemotherapy*, *65*(8), 1642–1645. https://doi.org/10.1093/jac/dkq167

Bielen, A., Šimatović, A., Kosić-Vukšić, J., Senta, I., Ahel, M., Babić, S., Jurina, T., González Plaza, J. J., Milaković, M., & Udiković-Kolić, N. (2017). Negative environmental impacts of antibiotic-contaminated effluents from pharmaceutical industries. *Water Research*, *126*, 79–87. https://doi.org/10.1016/j.watres.2017.09.019

Booth, A., Aga, D. S., & Wester, A. L. (2020). Retrospective analysis of the global antibiotic residues that exceed the predicted no effect concentration for antimicrobial resistance in various environmental matrices. *Environment International*, *141*, 105796. https://doi.org/10.1016/j.envint.2020.105796

Deng, W., Li, N., Zheng, H., & Lin, H. (2016). Occurrence and risk assessment of antibiotics in river water in Hong Kong. *Ecotoxicology and Environmental Safety*, *125*, 121–127. https://doi.org/10.1016/j.ecoenv.2015.12.002

Dinh, Q., Moreau-Guigon, E., Labadie, P., Alliot, F., Teil, M.-J., Blanchard, M., Eurin, J., & Chevreuil, M. (2017). Fate of antibiotics from hospital and domestic sources in a sewage network. *Science of The Total Environment*, *575*, 758–766. https://doi.org/10.1016/j.scitotenv.2016.09.118

Dinh, Q. T., Alliot, F., Moreau-Guigon, E., Eurin, J., Chevreuil, M., & Labadie, P. (2011). Measurement of trace levels of antibiotics in river water using on-line enrichment and triple-quadrupole LC–MS/MS. *Talanta*, *85*(3), 1238–1245. https://doi.org/10.1016/j.talanta.2011.05.013

Fernando, D. M., Xu, W., Loewen, P. C., Zhanel, G. G., & Kumar, A. (2014). Triclosan Can Select for an AdeIJK-Overexpressing Mutant of Acinetobacter baumannii ATCC 17978 That Displays Reduced Susceptibility to Multiple Antibiotics. *Antimicrobial Agents and Chemotherapy*, *58*(11), 6424–6431. https://doi.org/10.1128/aac.03074-14

Heuer, H., & Smalla, K. (2007). Manure and sulfadiazine synergistically increased bacterial antibiotic resistance in soil over at least two months. *Environmental Microbiology*, *9*(3), 657–666. https://doi.org/10.1111/j.1462-2920.2006.01185.x

Hricová, K., Röderová, M., Fryčák, P., Pauk, V., Kurka, O., Mezerová, K., Štosová, T., Bardoň, J., Milde, D., Kučová, P., & Kolář, M. (2021). Prevalence of Vancomycin-Resistant Enterococci and Antimicrobial Residues in Wastewater and Surface Water. *Life*, *11*(12), Article 12. https://doi.org/10.3390/life11121403

Hussein, M., Sun, Z., Hawkey, J., Allobawi, R., Judd, L. M., Carbone, V., Sharma, R., Thombare, V., Baker, M., Rao, G. G., Li, J., Holt, K. E., & Velkov, T. (2023). High-level nitrofurantoin resistance in a clinical isolate of Klebsiella pneumoniae: A

comparative genomics and metabolomics analysis. *mSystems*, *9*(1), e00972-23. https://doi.org/10.1128/msystems.00972-23

Karthikeyan, K. G., & Meyer, M. T. (2006). Occurrence of antibiotics in wastewater treatment facilities in Wisconsin, USA. *Science of The Total Environment*, *361*(1), 196–207. https://doi.org/10.1016/j.scitotenv.2005.06.030

Krantz, R. T., Nguyen, J. B., Renyer, K. M., Chiarelli, M. P., Hoellein, T. J., & Kelly, J. J. (2026). Adsorption of the antimicrobial triclosan to microplastics impacts biofilm and planktonic microbial communities in freshwater. *Science of The Total Environment*, *1012*, 181179. https://doi.org/10.1016/j.scitotenv.2025.181179

Looft, T., Johnson, T. A., Allen, H. K., Bayles, D. O., Alt, D. P., Stedtfeld, R. D., Sul, W. J., Stedtfeld, T. M., Chai, B., Cole, J. R., Hashsham, S. A., Tiedje, J. M., & Stanton, T. B. (2012). In-feed antibiotic effects on the swine intestinal microbiome. *Proceedings of the National Academy of Sciences*, *109*(5), 1691–1696. https://doi.org/10.1073/pnas.1120238109

Majeed, H. J., Riquelme, M. V., Davis, B. C., Gupta, S., Angeles, L., Aga, D. S., Garner, E., Pruden, A., & Vikesland, P. J. (2021). Evaluation of Metagenomic-Enabled Antibiotic Resistance Surveillance at a Conventional Wastewater Treatment Plant. *Frontiers in Microbiology*, *12*. https://www.frontiersin.org/articles/10.3389/fmicb.2021.657954

Miossec, C., Claudon, M., Levasseur, P., & Black, M. T. (2013). The β-lactamase inhibitor avibactam (NXL104) does not induce ampC β-lactamase in Enterobacter cloacae. *Infection and Drug Resistance*, *6*, 235–240. https://doi.org/10.2147/IDR.S53874

Silva, K. P. T., Sundar, G., & Khare, A. (2023). Efflux pump gene amplifications bypass necessity of multiple target mutations for resistance against dual-targeting antibiotic. *Nature Communications*, *14*(1), Article 1. https://doi.org/10.1038/s41467-023-38507-4

Sutcliffe, J., Grebe, T., Tait-Kamradt, A., & Wondrack, L. (1996). Detection of erythromycin-resistant determinants by PCR. *Antimicrobial Agents and Chemotherapy*, *40*(11), 2562–2566. https://doi.org/10.1128/aac.40.11.2562

Tahrani, L., Van Loco, J., Ben Mansour, H., & Reyns, T. (2015). Occurrence of antibiotics in pharmaceutical industrial wastewater, wastewater treatment plant and sea waters in Tunisia. *Journal of Water and Health*, *14*(2), 208–213. https://doi.org/10.2166/wh.2015.224

Watkinson, A. J., Murby, E. J., & Costanzo, S. D. (2007). Removal of antibiotics in conventional and advanced wastewater treatment: Implications for environmental discharge and wastewater recycling. *Water Research*, *41*(18), 4164–4176. https://doi.org/10.1016/j.watres.2007.04.005

Xu, J., Xu, Y., Wang, H., Guo, C., Qiu, H., He, Y., Zhang, Y., Li, X., & Meng, W. (2015). Occurrence of antibiotics and antibiotic resistance genes in a sewage treatment

plant and its effluent-receiving river. *Chemosphere*, *119*, 1379–1385. https://doi.org/10.1016/j.chemosphere.2014.02.040

Yoneda, K., Chikumi, H., Murata, T., Gotoh, N., Yamamoto, H., Fujiwara, H., Nishino, T., & Shimizu, E. (2005). Measurement of Pseudomonas aeruginosa multidrug efflux pumps by quantitative real-time polymerase chain reaction. *FEMS Microbiology Letters*, *243*(1), 125–131. https://doi.org/10.1016/j.femsle.2004.11.048
